# Microenvironment Restruction of Emerging 2D Materials and their Roles in Therapeutic and Diagnostic Nano‐Bio‐Platforms

**DOI:** 10.1002/advs.202207759

**Published:** 2023-05-02

**Authors:** Qian Li, Xizheng Wu, Shengdong Mu, Chao He, Xiancheng Ren, Xianglin Luo, Mohsen Adeli, Xianglong Han, Lang Ma, Chong Cheng

**Affiliations:** ^1^ College of Polymer Science and Engineering State Key Laboratory of Polymer Materials Engineering Department of Ultrasound West China Hospital Sichuan University Chengdu 610065 China; ^2^ Department of Organic Chemistry Faculty of Chemistry Lorestan University Khorramabad 68137‐17133 Iran; ^3^ Department of Chemistry and Biochemistry Freie Universität Berlin Takustrasse 3 14195 Berlin Germany; ^4^ State Key Laboratory of Oral Diseases National Clinical Research Center for Oral Diseases West China Hospital of Stomatology Sichuan University Chengdu 610041 China

**Keywords:** 2D materials, biofunctionalities, biomedical applications, microenvironments restruction, nano‐bio‐platforms

## Abstract

Engineering advanced therapeutic and diagnostic nano‐bio‐platforms (NBPFs) have emerged as rapidly‐developed pathways against a wide range of challenges in antitumor, antipathogen, tissue regeneration, bioimaging, and biosensing applications. Emerged 2D materials have attracted extensive scientific interest as fundamental building blocks or nanostructures among material scientists, chemists, biologists, and doctors due to their advantageous physicochemical and biological properties. This timely review provides a comprehensive summary of creating advanced NBPFs via emerging 2D materials (2D‐NBPFs) with unique insights into the corresponding molecularly restructured microenvironments and biofunctionalities. First, it is focused on an up‐to‐date overview of the synthetic strategies for designing 2D‐NBPFs with a cross‐comparison of their advantages and disadvantages. After that, the recent key achievements are summarized in tuning the biofunctionalities of 2D‐NBPFs via molecularly programmed microenvironments, including physiological stability, biocompatibility, bio‐adhesiveness, specific binding to pathogens, broad‐spectrum pathogen inhibitors, stimuli‐responsive systems, and enzyme‐mimetics. Moreover, the representative therapeutic and diagnostic applications of 2D‐NBPFs are also discussed with detailed disclosure of their critical design principles and parameters. Finally, current challenges and future research directions are also discussed. Overall, this review will provide cutting‐edge and multidisciplinary guidance for accelerating future developments and therapeutic/diagnostic applications of 2D‐NBPFs.

## Graphical abstract

Recent advancements toward creating advanced nano‐bio‐platforms via emerging 2D materials (2D‐NBPFs) are comprehensively summarized with unique insights into the corresponding molecularly restructured microenvironments and biofunctionalities. The synthetic strategies, recent key achievements in tuning biofunctionalities, and representative therapeutic and diagnostic applications are thoroughly discussed. This review provides cutting‐edge and multidisciplinary guidance for accelerating future developments and therapeutic/diagnostic applications of 2D‐NBPFs.

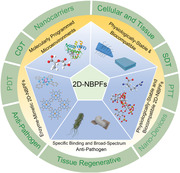

## 1 Introduction

Recently developed nanomaterials‐based therapeutic and diagnostic nano‐bio‐platforms (NBPFs) have resulted in new horizons for creating new pathways and breakthroughs against a wide range of challenging diseases,^[^ ^1^, ^2^, ^3^, ^4^, ^5^ ^]^ for instance, cancer therapeutics, pathogen inhibition, tissue regeneration, cellular and tissue imaging, biosensing nanodevices, and corresponding combination therapies (e.g., engineered chemotherapies with immunotherapy).^[^ ^6^, ^7^, ^8^, ^9^, ^10^ ^]^ Although significant progress has been made, most of these NBPFs serve as nanocarriers and present limited biofunctionalities or bioactivities. Key reasons for this lack of biofunctionalities and bioactivities at the nano‐bio‐interfaces include small surface areas, poor conductivity, low photo‐to‐thermal conversion efficiency, insufficient bioadhesiveness, rarely enzyme‐mimetic properties, etc.^[^ ^11^ ^]^ To overcome these challenges, researchers from multidisciplinary fields should work together to extend the boundaries of nanomaterials‐based therapeutics and diagnostics, which have inspired abundant new findings in both fundamental research and application investigations.^[^ ^12^, ^13^, ^14^, ^15^, ^16^ ^]^

In the past few years, tremendous endeavors have been inputted in engineering advanced therapeutic and diagnostic NBPFs with tunable bio‐functionalities and bioactivities.^[^ ^6^, ^17^, ^18^ ^]^ Among them, the emerging 2D materials have attracted extensive scientific interest as fundamental building blocks or nanostructures among material scientists, chemists, biologists, and doctors because of their advantageous physicochemical and biological properties. The family of emerging 2D materials mainly includes 2D metal–organic frameworks (MOFs),^[^ ^5^, ^19^, ^20^, ^21^ ^]^ 2D covalent organic frameworks (COFs),^[^ ^22^, ^23^, ^24^, ^25^, ^26^ ^]^ graphitic carbon nitride (g‐C_3_N_4_),^[^ ^27^, ^28^, ^29^ ^]^ black phosphorus (BP),^[^ ^30^, ^31^ ^]^ layered double hydroxides (LDH),^[^ ^32^, ^33^ ^]^ MXenes,^[^ ^34^, ^35^ ^]^ transition‐metal chalcogenides/dichalcogenides (TMCs/TMDCs),^[^ ^36^, ^37^ ^]^ etc.; but it does not involve graphene‐ or carbon‐based nanomaterials. Compared with conventional nanomaterials, the emerging 2D materials offer distinct advantages such as 1) the sheet‐like 2D structure with atomic thickness offers them better flexibility, large surface area, and facile modification availability^[^ ^38^, ^39^, ^40^, ^41^, ^42^ ^]^; 2) tunable bonding states and band structures between layers^[^ ^37^, ^43^, ^44^ ^]^; 3) superior chemical and physical properties, including photothermal, photodynamic, electrochemical, optical, biocatalytic, etc.^[^ ^45^, ^46^, ^47^, ^48^ ^]^ These characteristics and advantages have driven researchers to create abundant therapeutic and diagnostic NBPFs via the emerging 2D materials (2D‐NBPFs) in diverse biomedical fields.^[^ ^6^, ^23^, ^42^, ^49^, ^50^, ^51^ ^]^

The critical points for designing efficient 2D‐NBPFs include i) the physiological stability and biocompatibility; ii) the bioadhesiveness and surface functional groups; iii) molecularly programmed specific binding ability and stimuli‐responsiveness; iv) the active centers/adsorption sites and the interfacial microenvironments of carrier materials. Regulating and understanding the biofunctionalities and interface microenvironments of 2D‐NBPFs are unfading topics in nanomedicines. However, accompanying the prospects, there are nonetheless significant challenges in the synthesis, characterization, analysis, and clinical applications of 2D‐NBPFs due to the large diversity of functional moieties and support materials.^[^ ^52^, ^53^, ^54^ ^]^ In recent years, there are some reviews have summarized the biomedical applications of these emerging 2D‐NBPFs, including applications in drug delivery, cancer therapy, tissue engineering, bioimaging, and biosensing.^[^ ^45^, ^46^, ^48^ ^]^ However, few of them pay attention to engineering and analyzing the complex molecularly restructured microenvironments and biofunctionalities of 2D‐NBPFs that have well‐defined chemical structures or configurations.^[^ ^47^, ^53^, ^55^, ^56^, ^57^ ^]^

**Scheme 1:**
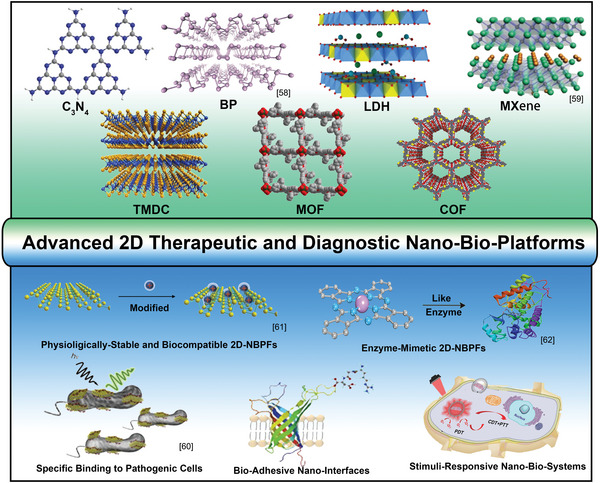
Chemical structures of emerging 2D materials and design of NBPFs for therapeutic and diagnostic applications. BP: Reproduced with permission.^[^ ^58^ ^]^ Copyright 2019, Elsevier. MXene: Reproduced with permission.^[^ ^59^ ^]^ Copyright 2018, American Chemical Society. Specific binding to Pathogenic cells: Reproduced with permission.^[^ ^60^ ^]^ Copyright 2018, Springer Nature. Physiologically‐stable and biocompatible 2D‐NBPFs. Reproduced with permission.^[^ ^61^ ^]^ Copyright 2017, American Chemical Society. Enzyme‐Mimetic 2D‐NBPFs. Reproduced with permission.^[^ ^62^ ^]^ Copyright 2022, American Chemical Society.

To direct the future engineering of 2D‐NBPFs, this timely review aims to offer a multidisciplinary summarization of modulating the molecularly restructured microenvironments and disclosing the structure–property correlations of diverse biofunctionalities in 2D‐NBPFs (**Scheme** **1**). First, we focus on an up‐to‐date overview of the synthetic strategies for designing 2D‐NBPFs with a cross‐comparison of their advantages and disadvantages. After that, we summarize the recent key achievements in tuning the functionalities of 2D‐NBPFs via molecularly programmed microenvironments, including physiological stability, biocompatibility, bioadhesiveness, specific binding to pathogens, broad‐spectrum pathogen inhibitors, stimuli‐responsive systems, and enzyme‐mimetics. Moreover, the representative therapeutic and diagnostic applications of 2D‐NBPFs have also been discussed with detailed disclosure of their critical design principles and parameters. Finally, current challenges and future research directions are also discussed. Overall, due to the lack of comprehensive summarization, this review will provide cutting‐edge and multidisciplinary guidance for accelerating future developments and therapeutic/diagnostic applications of 2D‐NBPFs across broad fields.

## 2 Synthesis and Physicochemical Properties of NBPFs via Emerging 2D Materials (2D‐NBPFs)

**Scheme 2:**
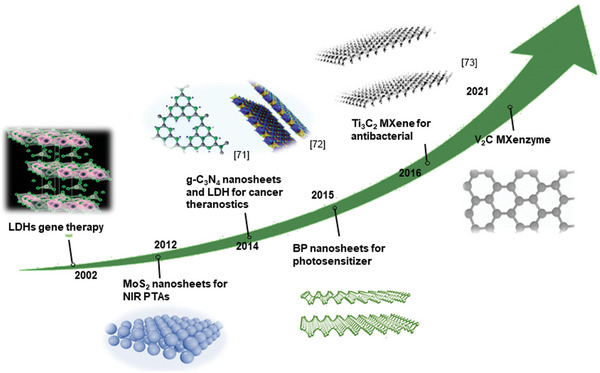
A timeline of important milestones in the development of 2D‐NBPFs. g‐C_3_N_4_: Reproduced with permission.^[^ ^71^ ^]^ Copyright 2018, American Chemical Society. LDH: Reproduced with permission.^[^ ^72^ ^]^ Copyright 2021, Springer Nature. MXene: Reproduced with permission.^[^ ^73^ ^]^ Copyright 2019, The Royal Society of Chemistry.

Over the last dozen years, significant progress has been made in studying 2D‐NBPFs, which has attracted extensive scientific interest and greatly enriched the variety of 2D‐NBPFs.^[^ ^63^, ^64^, ^65^, ^66^, ^67^ ^]^ As shown in **Scheme** **2**, in 2002, the LDHs had already been utilized as a potential gene delivery carrier for nanotherapeutics. In 2008, after Liu et al. for the first‐time discovered nanographene as a stable carrier for delivering the insoluble drug, the emerging 2D materials have undergone exponential studies to serve as NBPFs in versatile bioapplications, such as MoS_2_ nanosheets for photothermal nanoagents, MXene for antibacterial materials, and very recently the establishment of V_2_C MXenzyme.^[^ ^68^ ^]^ Due to their large surface area and physical/chemical effect arising from the atomically thin layer, and unique biological tunability, the 2D‐NBPFs have achieved impressive advances in nanomedicines and biomedical fields.

In particular, the adjustability is reflected in the fact that 2D‐NBPFs can regulate advanced therapeutic and diagnostic effects by changing their shapes, surface chemistries, thicknesses, and sizes.^[^ ^69^, ^70^ ^]^ Hence, the structure of 2D‐NBPFs must be carefully considered and fully controlled for biomedical applications. In this part, the synthesis strategies for a wide range of emerging 2D‐NBPFs series will be systematically summarized. Meanwhile, the essential structures and some featured examples of biomedical applications of 2D‐NBPFs will also be briefly discussed with a cross‐comparison of their advantages and disadvantages.

### 2.1 General Synthetic Strategies of 2D‐NBPFs

Thin 2D‐NBPFs can produce various physicochemical properties and stimulate different synthesis strategies. Here, we mainly discuss two synthetic methods: 1) top‐down synthesis and 2) bottom‐up synthesis. Both techniques with unique synthetic mechanisms have been extensively developed.

#### 2.1.1 Top‐Down Strategy

The top‐down method is a general strategy for the preparation of 2D‐NBPFs, where extensively exfoliated bulk 2D crystals are directly fabricated into thin‐layer 2D nanosheets by external forces.^[^ ^74^, ^75^, ^76^, ^77^ ^]^ This method is only applicable to bulk crystals such as TMDCs, BP, MOF/COF, and MAX phase‐based materials. These bulk crystals are driven by external forces to obtain thin‐layered 2D‐NBPFs by exploiting weak interlayer interactions.^[^ ^1^, ^78^ ^]^ With the development of science and technology, all kinds of synthetic methods have been employed to strip 2D‐NBPFs, for example, mechanical exfoliation, liquid phase stripping, and the electrochemical intercalation/exfoliation method; most of them have been successfully extended to synthesize bulk 2D crystals.^[^ ^51^, ^78^, ^79^, ^80^ ^]^ Mechanical exfoliation way has been verified as a simple and convenient process to accomplish stripping of different bulk crystals without participating in chemical reactions. The method includes two auxiliary stripping approaches: ultrasound‐assisted stripping and shear force‐assisted liquid‐phase stripping.

**Figure 1:**
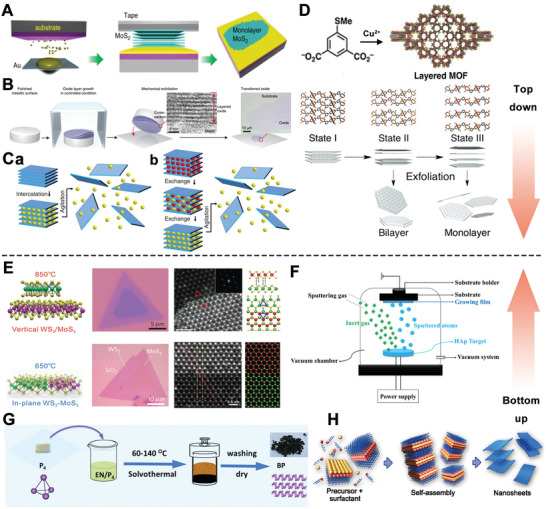
A) Illustrative images of the exfoliation process. Reproduced with permission.^[^ ^81^ ^]^ Copyright 2020, Springer Nature. B) A schematic illustration of layered growth and mechanical exfoliation. Reproduced with permission.^[^ ^94^ ^]^ Copyright 2021, Springer Nature. C) The schematic image of ion intercalation‐assisted and ion exchange‐assisted exfoliation. Reproduced with permission.^[^ ^95^ ^]^ Copyright 2022, Elsevier. D) Scheme of preparation of a layered MOF. Reproduced with permission.^[^ ^85^ ^]^ Copyright 2019, American Chemical Society. E) Illustrative image of in‐plane WS_2_‐MoS_2_ heterostructure formation at high temperatures with 650 and 850 °C. Reproduced with permission.^[^ ^90^ ^]^ Copyright 2014, Springer Nature. F) A schematic image of PVD process. Reproduced with permission. Reproduced with permission.^[^ ^96^ ^]^ Copyright 2021, Multidisciplinary Digital Publishing Institute. G) The schematic of BP solvothermal preparation.^[^ ^97^ ^]^ Copyright 2018, the National Academy of Sciences. H) Illustrative images of the self‐assembly process. Reproduced with permission.^[^ ^98^ ^]^ Copyright 2017, Springer Nature.

Although these stripping methods endow the exfoliated 2D‐NBPFs with apparent flat morphology and large surface size, their low controllability makes it the 2D‐NBPFs difficult to achieve large‐scale preparation. And liquid‐phase stripping process exfoliates bulk material through jet cavitation in liquid media, including organic solvents, polymer, and aqueous surfactant solutions, .c. Thus, it could induce the poison effect due to these organic solvents. Recently, in work exploited by Huang and colleagues,^[^ ^81^ ^]^ BP and TMDCs could be prepared via the Au‐assisted mechanical exfoliation method (**Figure** **1**A), and this reactive process is suitable for producing large‐area thin layers. Hence, it supports research of fundamental properties and practical application of 2D‐NBPFs. Moreover, as shown in Figure 1B, the even growth of layered metal oxides was prepared at the metal–air interface, which achieved nanometer polishing of large metal surfaces and defect drive oxidation promotion. After the 2D layered oxide structure is formed, the polished metal surface is punched onto the desired substrate for mechanical exfoliation of single and minority atomic layers.

**Figure 2:**
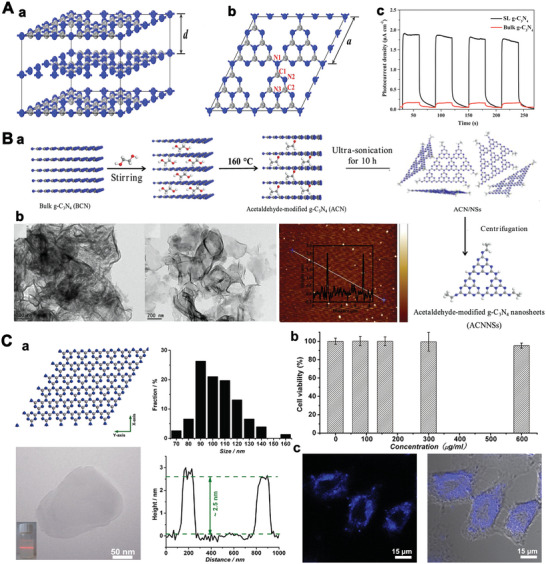
A) Structure model of (a) bulk g‐C_3_N_4_ and (b) Single‐layer g‐C_3_N_4_. Reproduced with permission.^[^ ^124^ ^]^ Copyright 2017, Elsevier. (c) The relationship between photocurrent and irradiation time of bulk g‐C_3_N_4_ and SL‐g‐C_3_N_4_ electrodes. Reproduced with permission.^[^ ^125^ ^]^ Copyright 2014, Elsevier. B(a)) Schematic illustrative of the synthetic process of ultrathin nanosheets from g‐C_3_N_4_. (b) Transmission electron microscope (TEM) images of g‐C_3_N_4_ and acetaldehyde‐modified g‐C_3_N_4_, and atomic force microscope (AFM) images of ACNNSs. Reproduced with permission.^[^ ^122^ ^]^ Copyright 2018, American Chemical Society. C(a)) Structure illustrative of g‐C_3_N_4_. TEM image and size distribution of the ultrathin g‐C_3_N_4_ nanosheet. (b) Cell viability of cultivating with 2D g‐C_3_N_4_ nanosheets. (c) Confocal fluorescence image. Reproduced with permission.^[^ ^126^ ^]^ Copyright 2013, The Royal Society of Chemistry.

Besides, layer nanomaterials containing ions or molecules between the layers were exfoliated by ion intercalation‐assisted stripping (**Figure** **2**C(a)), ion exchange‐assisted stripping (Figure 2C(b)), and chemical etching. Removal of specific layered materials, including oxides and hydroxides, can be achieved by ion exchange‐induced intercalation. If there is an electrostatic attraction between the layers, which can make them difficult to exfoliate with mechanical assistance.^[^ ^82^ ^]^ Therefore, the exfoliation of these 2D layered NBPFs can be achieved by increasing the interlayer spacing to weaken the interaction. However, there are still several problems, such as the time‐consuming and smaller lateral size. Besides, layer oxides, LDHs, and MAX phase nanomaterials cannot be stripped via the ion exchange method because of the strong bonding between ions and the host layers, some of which are strong covalent bonds. Therefore, a chemical reaction with a more potent force can break the bonding. Currently, the chemical etching method has been used to exfoliate MAX phase nanomaterials and made great progress in preparing various MAX phases. For example, Michael et al.^[^ ^83^ ^]^ synthesized 2D transition metal carbides and carbonitrides via steeping MAX phase powders (Ti_2_AlC, Ta_4_AlC_3_, Ti_3_AlCN, (V_0.5_, Cr_0.5_)_3_AlC_2_, and (Ti_0.5_, Nb_0.5_)_2_AlC) in hydrofluoric acid (HF).

Compared with the bulk 2D crystals, the stripped thin sheet has extra‐small size, abundant surface functional, groups ultrahigh specific surface, and defect sites. These characteristics are beneficial for further surface modification via DNA, molecular medicine, protein, and other small functional materials. For instance, 2D TMDCs were exfoliated by regulation of the hydrophobic interaction between poly(*ε*‐caprolactone)‐*b*‐poly(ethylene glycol) (PCL‐*b*‐PEG) and the surface of TMDCs.^[^ ^84^ ^]^ In terms of 2D‐MOF, thin‐layer porous MOFs can provide regular and ordered nanopore arrangement. In Figure 1D, MOF crystals stepwise expansion followed by exfoliation to prepare 2D ultrathin porous nanosheets.^[^ ^85^ ^]^

With high surface area and anisotropic physicochemical properties, 2D MOF nanosheets exhibit different biological behaviors from 3D materials. Notably, thin‐layer materials synthesis by reducing the dimensionality of nanomaterials may offer a potential opportunity to study the distinctive biological behavior of 2D nanomaterials. The 2D‐MOF nanosheet exhibits distinct bio‐behaviors from 3D materials because of their large specific surface areas and particular physicochemical and biomedical properties.^[^ ^86^, ^87^ ^]^ Remarkably, thin‐layer nanomaterials formed by reducing the dimensionality of crystal materials may offer a great opportunity to study low‐dimensional nanomaterials with unique biological properties.

#### 2.1.2 Bottom‐Up Synthesis

The bottom‐up synthesis strategy is an alternative to the top‐down synthesis of 2D‐NBPFs. The synthesis strategy can control the morphology features and chemical compositions with better optimization from the molecular level.^[^ ^88^ ^]^ It includes chemical vapor deposition (CVD), and physical vapor deposition (PVD). The most common method is the CVD growth strategy, which has advantages for the synthesis of 2D‐NBPFs with high quality, controllable morphology, and stable dimensions. Recently, it has been found that hexagonal boron nitride and transition metal dichalcogenides can also be synthesized by applying the 2D diffusion‐limited aggregation model and CVD synthesis method, which achieves the precise control of CVD growth of 2D materials. Besides, the WS_2_ nanosheets can be fabricated with synchronous surface modification for medical imaging via the CVD way.^[^ ^89^ ^]^ Obviously, the CVD method establishes a convenient and straightforward route to prepare various fractal‐morphology high‐quality 2D materials.

Physical synergy is an effective approach to enhance the controllability of CVD growth. In addition, the CVD process is also closely related to the temperature pressure and growth time of the reaction itself. Figure 1E shows a schematic image of the heterostructures, which were forming due to the different nucleation rates of MoS_2_ and WS_2_ at 650 and 850 °C.^[^ ^90^ ^]^ Hence, the product of an in‐plane WS_2_‐MoS_2_ heterostructure or the thermodynamic product of a WS_2_/MoS_2_ bimolecular layer can be selectively obtained with this method by adjusting the temperature. In summary, high‐quality 2D‐NBPFs with excellent stability could be fabricated using a bottom‐up approach. However, the biomedical applications of CVD are limited by high temperatures and high vacuum conditions. PVD is also used to prepare 2D‐NBPFs (Figure 1F), including vacuum evaporation, sputtering coating, arc plasma coating, ion plating, molecular beam epitaxy, etc. The method also is limited by the strict vacuum conditions.

In addition, the method of wet chemical synthesis also belongs to the bottom‐up thought. Wet‐chemical hydro/solvothermal reaction (Figure 1G,H) is a course in which the formation and growth of crystals occur in a reaction still at high temperatures and pressures with reaction parameters of concentration, pressure, time, pH, and organic solvent.^[^ ^91^, ^92^, ^93^ ^]^ It is conducive to the formation of a complete crystal, uniform particle size distribution, and good dispersion of the synthesized products.

The structure and physicochemical properties of 2D‐NBPFs are affected by the synthesis methods and conditions. To facilitate the selection of suitable methods according to the practical application needs, we provide a comparative analysis and summary of the various synthetic methods (**Table** **1**). In addition, the structure and performance characteristics of materials are closely related to biomedical applications. Next, we carry out specific analysis of different 2D‐materials and summarize their characteristics in a **Table** **2**.

**Table 1:** Comparison of different preparation methods

Methods		Substrates types	Advantages	Disadvantages	Refs.
Top‐down strategy	Mechanical exfoliation	g‐C_3_N_4_ BP MXenes TMDCs COFs	Low cost, convenient preparation, and massive production	Uncontrollable size	[4, 74, 76, 81]
	Liquid phase stripping	g‐C_3_N_4_ BP TMDCs MOFs COFs	Low cost, fewer defects, and convenient preparation	Uncontrollable layer number	[4, 75, 77]
	Chemical etching	MXenes	Massive production and high quality	Narrow application range	[2, 83]
	Electrochemical/ion exfoliation/intercalation	LDH TMDCs MOFs	High‐quality and facile control on structures	High cost and complicated operation	[4, 80]
Bottom‐up synthesis	CVD	BP TMDCs	Large surface area growth, good crystallinity, and wide range of applications	High energy consumption	[88, 89]
	PVD	BP	Convenient preparation	Narrow application range	[96]
	Wet chemical synthesis	BP TMDCs MOFs	Convenient preparation	Uncontrollable layer number	[93, 97, 98]

**Table 2:** Comparison of different characteristic of 2D materials

Types of 2D material	Sample names	Characteristics	Refs.
Carbon nitride	g‐C_3_N_4_	Wide bandgap, fast carrier recombination, good photostability, good biocompatibility, and nontoxicity	[99]
BP	BP	Characteristics of switchable energy bands, high surface reactivity, and good biodegradability	[100, 101]
LDH	MnFe‐LDH CoMo‐LDH NiMo‐LDH CoW‐LDH MgFe‐LDH	Low‐dimensional layered crystal structure, the adjustable characteristics of internal and external surface properties, and low toxicity	[102, 103, 104, 105]
MXene	Ti_3_C_2_T*_x_* Ta_4_C_3_ V_2_CT*_x_*	Large specific surface area, and excellent photothermal conversion efficiency	[106, 107, 108]
TMDCs	MoS_2_ WS_2_	Adjustable structure, high electrical conductivity, and fast carrier recombination	[90, 109]
MOF	Zn_2_(PdTCPP) Hf‐Mn‐TCPP Ln‐TCPP TBP@MOL	A large specific surface, high porosity, controllable surface functionalization, and excellent biodegradability	[110, 111, 112, 113]
COF	Py‐Bpy TpASH	Designable Pore Structure And adjustable bandgap	[114, 115]

### 2.2 Carbon Nitrides‐Based 2D‐NBPFs

g‐C_3_N_4_ is a characteristic 2D crystal whose interlayer interaction of the bulk structure is van der Waals forces (Figure 2A(a)), which has been researched abundantly due to its excellent catalytic activity in the photo‐/electro‐field.^[^ ^116^, ^117^ ^]^ However, g‐C_3_N_4_ suffers from low conductivity, wide bandgap, fast carrier recombination, and few surface active sites, which have hindered its photocatalytic application.^[^ ^99^ ^]^ To improve the photocatalytic efficiency of g‐C_3_N_4_, g‐C_3_N_4_‐based NBPFs have been prepared by some modification methods, including atom doping, nanostructure control, introducing surface defects, etc.

The hexagonal carbon skeleton of N‐substituted carbon could define the configuration of single‐layer g‐C_3_N_4_ through sp^2^ hybridization of carbon and nitrogen atoms.^[^ ^118^, ^119^ ^]^ In the structure of g‐C_3_N_4_, three nonequivalent nitrogen atoms (donated as N1, N2, and N3) and two nonequivalent carbon atoms (donated as C1 and C2) could be noticed (Figure 2A(b)). The nonequivalent N_2_ atom is coordinated with the other two atoms (C1 and C2), and the remaining atoms are three‐coordinated.^[^ ^120^ ^]^ As shown in Figure 2A(c), single‐layer g‐C_3_N_4_ displays photocatalytic ability, showing an enhanced photocurrent. The separation of photogenerated charge could be further improved by the synergy of the low resistance and the short transfer distance.^[^ ^121^ ^]^ Meanwhile, defects were introduced into the g‐C_3_N_4_ structure via acetaldehyde modification (Figure 2B), which acted as an excitation energy trap and caused significant changes in fluorescence emission.^[^ ^122^ ^]^ Besides, introducing the aldehyde group also improves its water solubility. The prepared 2D g‐C_3_N_4_ with stable photoluminescence, good water solubility, and good biocompatibility exhibits selective fluorescence response to Ag^+^ in living cells. These factors might be to the benefit of carbon‐based with wider applications of nanomaterials as active materials for fluorescence detection. Besides, as shown in Figure 2C(a), the g‐C_3_N_4_ nanosheets were prepared by a “green” liquid ultrasonication and exfoliation.^[^ ^123^ ^]^ The obtained 2D g‐C_3_N_4_ nanosheets exhibited good acid‐base stability and pH‐dependent photoluminescence (Figure 2C(b,c)). Therefore, g‐C_3_N_4_ nanosheets can be used for fluorescence imaging due to their advantages of good photostability, good biocompatibility, and nontoxicity.

### 2.3 Black Phosphorus‐Based 2D‐NBPFs

**Figure 3:**
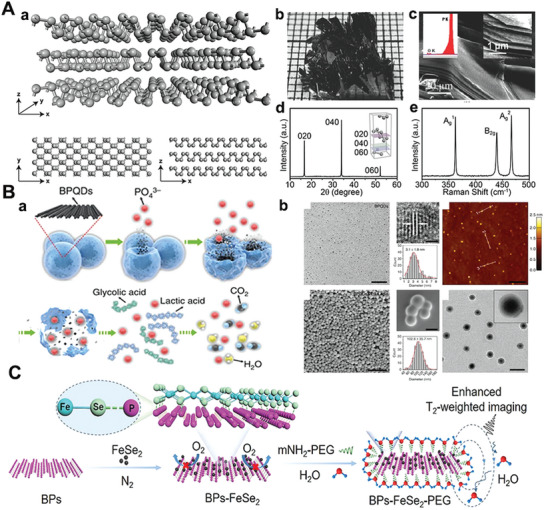
A(a) Structure illustration of three‐layer BP, 3D views, top view, and side view. Reproduced with permission.^[^ ^132^ ^]^ Copyright 2017, The Royal Society of Chemistry. (b) Optical diagram of BP. (c) SEM image of BP nanosheets. Reproduced with permission.^[^ ^130^ ^]^ Copyright 2018, American Society of Chemistry. (d) Diffraction of X‐rays (XRD) pattern of BP crystals. Inset: diagram of an orthogonal crystal BP. (e) Raman spectrum of the BP. Reproduced with permission.^[^ ^131^ ^]^ Copyright 2019, Frontiers in Chemistry. B(a)) Synthesis process of BP‐based nanospheres. (b) TEM, SEM, and AFM image of BP‐based nanospheres. Reproduced with permission.^[^ ^100^ ^]^ Copyright 2016, Springer Nature. C) The schematic of BPs‐FeSe_2_‐PEG heteronanostructure. Reproduced with permission.^[^ ^138^ ^]^ Copyright 2021, Springer Nature.

BP is one of the most stable allotropes of important 2D substances of phosphorus,^[^ ^127^ ^]^ and has emerged as an essential 2D‐NBPFs recently (**Figure** **3**A(a)).^[^ ^128^ ^]^ BP comprises corrugated phosphorus atoms with robust interlayer bonding and weak interlayer interaction.^[^ ^129^ ^]^ As shown in Figure 3A(b), the prepared BP crystals exhibit a divergent shape, with lots of sheet‐like branches, in the order of centimeters and black. At the same time, SEM images show that the BP crystal owns a layered structure (Figure 3A(c)). Furthermore, the XRD analysis shows three peaks of (020), (040), and (060) crystal planes in the range of 10°–60° (Figure 3A(d)),^[^ ^130^ ^]^ indicating that the BP crystal is an orthorhombic phase, and the crystal is highly oriented growth with preferable crystallinity. Moreover, three characteristic peaks caused by A_g_^1^, B_g_^2^, and A_g_^2^ modes in the rhombic BP lattice can be observed in the Raman spectrum (Figure 3A(e)).^[^ ^131^ ^]^ Single‐layered BP nanosheets can be obtained by mechanically exfoliating from bulk BP. As shown in Figure 3A(a), the bonding architecture of BP shows that two phosphorus atoms in adjacent planes are covalently bonded, and another P atom between adjacent planes through the p orbit is superimposed with the adjacent BP layers by a relatively weak Van der Waals interaction.^[^ ^132^ ^]^ Through sp^3^ track hybridization, BP exhibits a slightly crumpled honeycomb structure with armchairs and zigzag structures in the *x*‐axis and *y*‐axis directions, respectively.^[^ ^133^ ^]^

BP can efficiently load drug molecules, biomolecules, and antibodies due to the characteristics of switchable energy bands, high surface reactivity, and good biodegradability.^[^ ^100^, ^101^, ^134^, ^135^ ^]^ As shown in Figure 3B, 2D BP‐based NBPFs as a photothermal agent which was utilized in cancer theranostics with superior biocompatibility and showed excellent antitumor effects both in vitro and in vivo. Besides, because of the unique hexagonal honeycombs within the layer, BP nanosheets exhibit excellent light absorption performance and singlet oxygen generation properties.^[^ ^136^ ^]^ These features make the BP nanoplatform a great candidate for photodynamic therapy (PDT) or/and photothermal therapy (PTT) applications, which also helps construct a variety of collaborative treatments.^[^ ^136^, ^137^ ^]^

### 2.4 Layered Double Hydroxides‐Based 2D‐NBPFs

**Figure 4:**
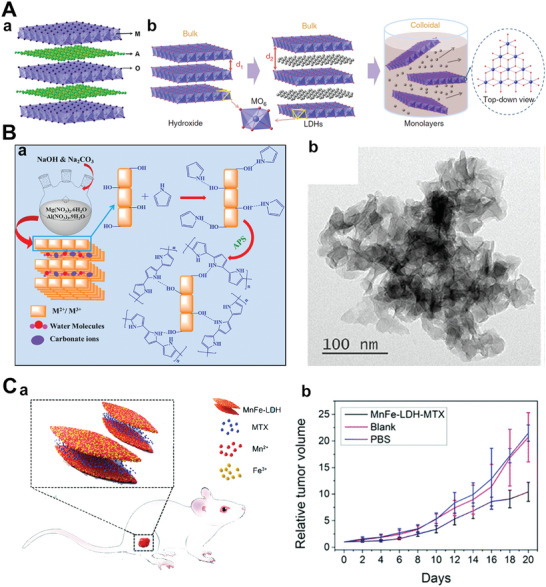
A(a)) The diagrammatic drawing of LDHs structure. Reproduced with permission.^[^ ^69^ ^]^ Copyright 2017, American Chemical Society. B(a)) Schematic image of the anion exchange‐assisted liquid exfoliation to obtain LDHs nanosheets. Reproduced with permission.^[^ ^149^ ^]^ Copyright 2014, Springer Nature. (b) TEM representation of MgAl LDH. Reproduced with permission.^[^ ^150^ ^]^ Copyright 2019, American Chemical Society. C) Relative tumor volume growth curves of S180 tumor‐bearing mice with PBS, Blank, and MnFe‐LDH‐MTX treatment. Reproduced with permission.^[^ ^102^ ^]^ Copyright 2017, The Royal Society of Chemistry.

LDHs are constituted by hydrotalcite‐like layered structures with a positive charge and an interlayer section comprising solvation molecules or anions.^[^ ^139^ ^]^ In particular, LDHs with a typical composition of [M_1−_ *_x_* ^2+^M*_x_* ^3+^(OH)_2_]*^x^* ^+^[A*_x_*_/_ *_n_*]*^n^* ^−^·mH_2_O are known as distinctive 2D compounds, where in most cases, M^2+^ (such as Mg^2+^, Zn^2+^, Ni^2+^) and M^3+^ (such as Ga^3+^, Al^3+^, Mn^3+^, Fe^3+^) are bivalent/trivalent metal cations, and A*^m^* ^−^ is an interlayer anion, respectively.^[^ ^140^, ^141^ ^]^ **Figure** **4**A,B illustrates the crystal structure of LDHs,^[^ ^69^ ^]^ where metal cations occupy the center of the octahedron, and the vertices of the octahedron contain OH^−^ ions. Moreover, these vertices could be connected to construct the 2D layer. The diversity of chemical composition, such as the interlayer anion (A*^n^* ^−^) and layer cations (M^2+^ or M^3+^), results in facile exfoliation properties and unique redox characteristics, which contributes notably to their pH‐induced drug release behaviors. Since the surface of LDH nanosheets is positively charged, LDHs can easily pass through negatively charged biomembranes and easily adsorb negatively charged drug molecules, and be released efficiently.^[^ ^142^, ^143^, ^144^ ^]^ As shown in Figure 4C, a negatively charged drug methotrexate (MTX) was loaded into the gallery of MnFe‐LDH,^[^ ^102^ ^]^ which can inhibit the growth of the solid tumor.

It is worth noting that LDHs can be absorbed by particular species of cancer cells through clathrin‐mediated endocytosis, consequently resulting in resistance to in vivo buffering.^[^ ^145^ ^]^ Due to these special properties, various studies have proved that negatively charged drugs are successfully delivered through LDHs, and the therapeutic effect is enhanced. Compared with other 2D‐NBPFs, LDHs have been extensively researched in the field of biomedicine, especially in the field of drug delivery, due to their low toxicity.^[^ ^146^, ^147^ ^]^ In addition, LDH has always been a suitable material for the development of nanocontrast agents because of the unique low dimensional layered crystal structure and the adjustable characteristics of internal and external surface properties.^[^ ^102^, ^148^ ^]^

### 2.5 MXenes‐Based 2D‐NBPFs

**Figure 5:**
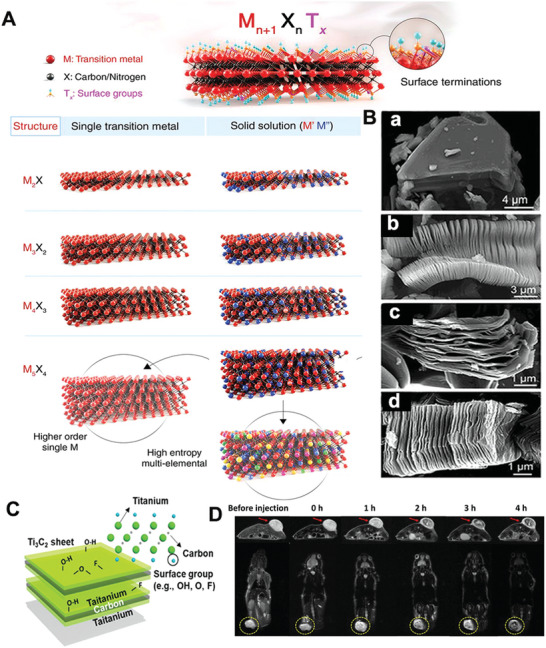
A) Composition of MXenes. Structure of MAX phases and the corresponding MXenes. Reproduced with permission,^[^ ^50^ ^]^ Copyright 2021, National Academy of Sciences. B) SEM images for (a) Ti_3_AlC_2_ before treatment, (b) Ti_3_C_2_, (d) Ta_4_C_3_, and (e) Ti_3_CN. Reproduced with permission.^[^ ^107^ ^]^ Copyright 2012, American Chemical Society. C) Schematic of MXene and its surface functional groups (—OH, —O, and —F). Reproduced with permission.^[^ ^160^ ^]^ Copyright 2020, Wiley‐VCH. D) Transverse and coronal MR images of soybean phospholipid‐modified Ta_4_C_3_T*_x_* before and after tumor treatment.^[^ ^161^ ^]^ Copyright 2020, Elsevier.

As an important member of the 2D‐NBPFs family, MXene is a single or few‐layer metallic carbide or nitride with surface terminations of O, OH, NH, F, Cl, et al.^[^ ^151^ ^]^ MXenes is synthesized by selective etching of “A” layers from the MAX phase precursor in strong acid,^[^ ^152^ ^]^ where “M” means transition metal (e.g., Ti, Nb, V, Cr, Mo, Sc, Zr), “A” represents layer atoms (e.g., Al, Si, Ga), and “X” indicates carbon and/or nitrogen (**Figure** **5**A).^[^ ^153^, ^154^ ^]^ The etching process results in MXene layers covered by functional groups of —O, —OH, —F, or —Cl, and the strong metal bond between M and A in the MAX phase precursor is replaced by the weak electrostatic interaction between M and A layers. The thin layer of M or A can be easily obtained by further sonication of the resulting products. MXene materials have a common formula, M*_n_*_+1_X*_n_* T*_x_*, where T*_x_* (*x* is the number of terminals) represents the functional groups, and *n* varies from 1 to 4.^[^ ^108^ ^]^ For instance, the chemical expression of a vanadium‐based MXene with two layers of the metal vanadium and unknown terminations would be V_2_CT*_x_*, and oxhydryl‐ or fluorine‐terminated V_2_CT*_x_* can be written as V_2_C(OH)_2_ or V_2_CF_2_, respectively. Moreover, if M sites are occupied by two transition metals in the MXene structure, the formula will be recorded as (M’, M’’)*_n_*_+1_X*_n_* T*_x_*, where M’ and M’’ are two different metals (Figure 5A). Figure 5B shows the SEM micrographs of different MXenes. HF is generally used to etch containing Al in MAX phases precursor, as shown in Figure 5B(b), which exhibits significant organ‐shape morphology.^[^ ^107^ ^]^ In addition, the method of chemical intercalation of organic molecules also are used to etch MAX phases, and then the prepared MXenes can be stripped into monolayer or few‐layer nanosheets (Figure 5B(c)).

Most MXene materials, such as Ti_3_C_2_T*_x_*,^[^ ^106^ ^]^ Nb_2_CT*_x_*,^[^ ^155^ ^]^ and Ta_4_C_3_T*_x_*,^[^ ^156^ ^]^ have good biocompatibility and low cytotoxicity. These materials have effectively shown surface plasmon resonance and high photothermal conversion efficiency in the near‐infrared (NIR) and infrared range, making MXene‐based 2D‐NBPFs promising for tumor diagnosis and treatment (Figure 5C). For example, soybean phospholipid‐modified Ta_4_C_3_T*_x_* were used for photothermal ablation of breast tumor cells,^[^ ^156^ ^]^ in which >90% of the tumor cells were killed by tumor cells cocultured with soybean phospholipid‐modified Ta_4_C_3_T*_x_* under NIR light. MXenes could also be used for drug delivery^[^ ^157^ ^]^ and magnetic resonance imaging (MRI)—imaging of tumors (Figure 5D).

Compared with graphene oxide, MXenes are targeted as good materials for membranes and implantable devices due to their higher resistance to bacterial attachment and accumulation.^[^ ^66^ ^]^ Besides, Ti_3_C_2_T*_x_* and Mo_2_TiC_2_T*_x_* can efficiently remove urea from the dialysate, outperforming the conventional sorbents, for instance, wearable artificial and treating COVID‐19.^[^ ^158^, ^159^ ^]^ Therefore, MXene has been considered a promising candidate for the wearable artificial kidney. The charged functional groups at the MXene surface act as active adsorption sites for toxins, and the gaps between MXene sheets result in ultrahigh adsorption efficiency.

### 2.6 Transition‐Metal Chalcogenides/Dichalcogenides‐Based 2D‐NBPFs

**Figure 6:**
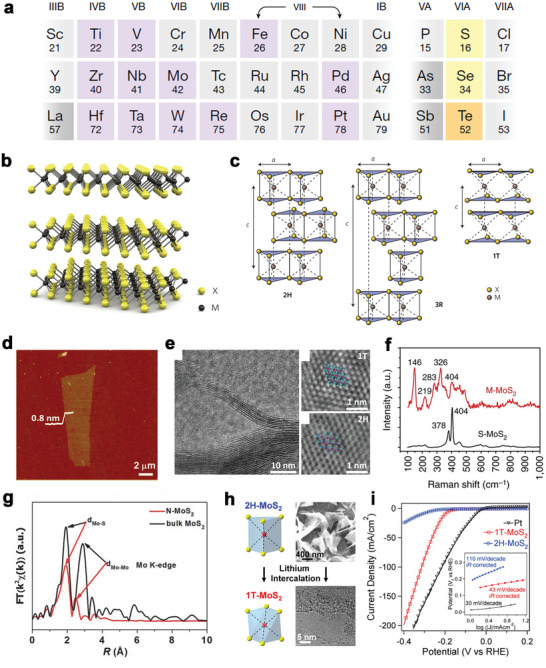
A) Composition of TMDCs. Reproduced with permission.^[^ ^162^ ^]^ Copyright 2018, Springer Nature. B) Structural model of a typical MX_2_. C) The structural polytypes of TMDCs: 2H, 3R, and 1T. Reproduced with permission.^[^ ^165^ ^]^ Copyright 2012, Springer Nature. D) AFM image of single‐layer MoS_2_ nanosheet. Reproduced with permission.^[^ ^167^ ^]^ Copyright 2012, American Chemical Society. E) TEM images of 1T‐(right‐top) and 2H‐(right‐bottom) MoS_2_. Reproduced with permission.^[^ ^168^ ^]^ Copyright 2020, Wiley‐VCH. F) Raman shift of M‐MoS_2_ and S‐MoS_2_. Reproduced with permission.^[^ ^169^ ^]^ Copyright 2016, Springer Nature. G) EXAFS spectra of Mo K‐edge in R‐space. Reproduced with permission.^[^ ^170^ ^]^ Copyright 2015, Wiley‐VCH. H) The conversion from 2H to 1T phase of MoS_2_ by Li‐intercalation. Reproduced with permission.^[^ ^174^ ^]^ Copyright 2013, American Chemical Society. I) The polarization curves of the 2H‐ and 1T‐ MoS_2_. Inset: Tafel slopes of 2H‐ and 1T‐ MoS_2_. Reproduced with permission.^[^ ^173^ ^]^ Copyright 2017, American Chemical Society.

TMCs/TMDCs are a series of compounds with a common composition of MX_2_, where M represents the metal element in group IVB, VB, and VIB, and X indicates a chalcogen element as shown in **Figure** **6**A.^[^ ^162^, ^163^, ^164^ ^]^ The formation of X–M–X can describe the layered structure of TMDCs, and the X atom with the form of hexagonal planes is separated by metal atom planes (Figure 6B(a)).^[^ ^165^ ^]^ Adjacent layers could be weakly connected through the van der Waals force, thereby forming the bulk crystal with multiple polytypes. There are three typical crystal structures of TMDCs (1T, 2H, and 3R). The overall symmetry of TMDCs in the 1T phase is hexagonal, and the metal atoms have octahedral prismatic coordination (Figure 6B(c)), while the 2H or 3R structure is coordinated by the same metal atom possesses trigonal prismatic phase. The single‐layer 2D TMDCs, which inherit the crystal structure of the bulk materials, can be obtained by exfoliation from the bulk counterpart via different driving forces.

In Figure 6C, MoS_2_ produced via the scotch‐tape‐based mechanical exfoliation method shows ultrathin flakes of 0.8 nm thickness.^[^ ^166^, ^167^ ^]^ As for the phases of 2D TMDCs, due to the unique features, the metallic 1T phase can be separated from the original 2H phase. The 1T and 2H structures of MoS_2_ are shown in the top view of the atomic resolution STEM images of few‐layered 2D MoS_2_ nanosheets (Figure 6D).^[^ ^168^ ^]^ The single‐layer 1T phase MoS_2_ exhibits an octahedral lattice, where each Mo atom is enclosed by six S atoms, while three S atoms surround the Mo atom of the 2H phase MoS_2_ in the trigonal phase. Figure 6E shows the Raman spectra of two typical phases of MoS_2_, which tells the essential difference between the 1T and 2H phases.^[^ ^169^ ^]^ The Mo—Mo stretching vibrations in 1T MoS_2_ resulted in a strong Raman band at 146 cm^−1^_,_ while 2H MoS_2_ presents characteristic peaks of ≈378 and 404 cm^−1^ for E^[^ ^1^ ^]^_2 g_ and A_1g_. The Fourier‐transform profiles of the EXAFS data on the Mo K‐edge of R space (Figure 6F) reveal a significant change in the second peak linked to the Mo—Mo bond from 3.18 to 2.75 Å,^[^ ^170^ ^]^ indicating the 2H to 1T structure transformation further shortens the Mo—Mo bond of MoS_2_ (Figure 6I). Moreover, the coordination number of the Mo atom decreased, which could be observed in the reduction of the closest Mo—Mo band intensity. These phenomena demonstrate that 1T MoS_2_ with distorted octahedral coordination can serve as 2D‐NBPFs for practical use.

Recently, 2D TMDCs have been applied in the biomedical field. TMDCs can immobilize a large number of biomolecules per unit area and can be used for efficient biosensor design to detect various analytes, such as DNA, glucose, dopamine, and some reactive oxygen species.^[^ ^49^, ^171^, ^172^ ^]^ As shown in Figure 6G, a simple and convenient electrochemical sensing platform was prepared to probe microRNA‐21 with high sensitivity using a MoS_2_ nanosheet functionalized with thionine and gold nanoparticles.^[^ ^173^ ^]^

### 2.7 MOFs‐/COFs‐Based 2D‐NBPFs

**Figure 7:**
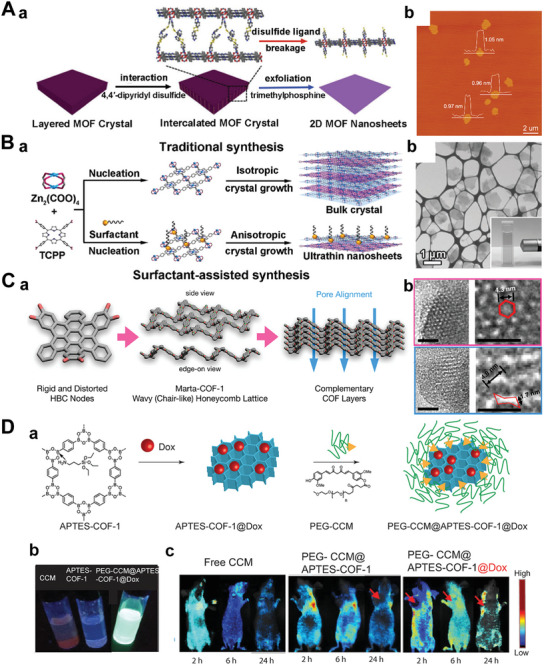
A(a)) Illustrative images of the synthesis process of 2D MOF. (b) AFM of 2D MOF nanosheets. Reproduced with permission.^[^ ^110^ ^]^ Copyright 2017, American Chemical Society. B(a)) Schematic images of the surfactant‐assisted method of 2D TCPP MOFs compared with the conventional nucleation method. (b) TEM image of TCPP(Zn) nanosheets. Reproduced with permission.^[^ ^180^ ^]^ Copyright 2015, Wiley‐VCH. C(a)) Schematic images of the conjugation of 2, 3, 10, 11, 18, 19‐hexahydroxy‐cata‐hexabenzocoronene in boronate COF to obtain Marta‐COF‐1. (b) High‐resolution transmission electron microscope (HRTEM) images of Marta‐COF‐1. Reproduced with permission.^[^ ^185^ ^]^ Copyright 2019, American Chemical Society. D) 2D COF‐based NBPFs (a) for the tumor targeting of chemotherapeutics (b,c). Reproduced with permission.^[^ ^55^ ^]^ Copyright 2018, Springer Nature.

MOFs are porous compounds that are self‐assembled and coordinated by inorganic metal nodes and organic ligands.^[^ ^175^, ^176^ ^]^ In a consequence of the adjustable structures, ultrahigh porosity, and high density and uniformly dispersed active sites, MOFs have attracted extensive attention in biomedical fields.^[^ ^177^, ^178^, ^179^ ^]^ What's more, the 3D MOFs with layered structures have been formed into 2D nanosheets. Both top‐down and bottom‐up methods could obtain 2D MOF nanosheets with sufficient active sites. Recently, constructing ordered 2D MOF nanosheets with tunable surface chemistry has attracted considerable interest. The interaction between the interlayers in MOFs with layered structures could be further weakened by intercalating other compounds. 2D MOF nanoflakes have been exfoliated from bulk MOF crystals (Zn_2_(PdTCPP) (TCPP = tetrakis (4‐carboxyphenyl)‐porphyrin)) by organic ligands intercalation and chemical exfoliation approach (**Figure** **7**A(a)).^[^ ^110^ ^]^ After synthesizing the layered MOF bulk, a 4,4′‐dipyridyl disulfide bond (DPDS) was employed to coordinate with metal nodes in the MOF crystal, thus weakening the interlayer interaction. Using trimethylphosphine to reduce disulfide bonds selectively, the single‐layer independent MOF nanosheets were prepared (Figure 7A(b)). Furthermore, the surfactant‐assisted synthetic method has also attracted researchers’ attention. The MOFs surface could be selectively fixed with the surfactant molecules, thereby controlling the formation of 2D MOF crystals. A class of ultrathin TCPP compounds coordinated with various metal nodes, such as Zn, Cu, Cd, and Co, have been prepared by this method (Figure 7B(a,b)).^[^ ^180^, ^181^ ^]^

COFs are porous molecular frameworks comprised of H elements and some of the second‐period elements, such as B, C, N, and O. These building atoms are connected by strong covalent bonds to form the ordered COF structure.^[^ ^181^ ^]^ Since the Yaghi group first reported the synthesis of COF in 2005,^[^ ^182^ ^]^ the research on COFs has aroused recent interest in various research fields. The robust covalent bonding of COFs leads to excellent thermal and chemical stability. The modular nature of these materials allows their pore geometries and chemical properties to be independently adjustable and can be leveraged to be efficient biomedical materials.^[^ ^3^, ^183^, ^184^ ^]^ Many properties of COFs strongly rely on the structural arrangements and constituents of the different layers. Designing the highly ordered 2D COFs to perform an enhanced catalytic activity is still a big challenge. To solve this issue, it has been proposed to use core‐twisted polycyclic aromatic hydrocarbon 2, 3, 10, 11, 18, 19‐hexahydroxy‐cata‐hexabenzocoronene as rigid nodes.^[^ ^185^ ^]^ 2, 3, 10, 11, 18, 19‐hexahydroxy‐cata‐hexabenzocoronene was locked in conformation due to the hyperemia of the space between the hydrogen on the surrounding benzene ring, with a twisted rigid structure (Figure 7C(a)). TEM revealed its chair‐like honeycomb facets and neatly arranged mesoporous channels (Figure 7C(b)). It is one of the most promising approaches to constructing diverse COF materials by forming the sp^2^‐carbon‐bonding pattern. Recently, the amine‐functionalized‐B‐O‐linkage‐based COF‐1 (APTES@COF‐1) has been prepared. Due to the poor water solubility of COF, it was modified with PEGylated curcumin, then underwent self‐assembly to form micelles encapsulating DOX (PEG‐CCM@APTES‐COF‐1), with good water solubility and dispersibility for drug delivery in the treatment of antitumor (Figure 7D(a,b)). As shown in Figure 7D(c), it exhibits intense fluorescence, which benefits tracking both in vitro cellular uptake research and in vivo tissue distribution. Finally, the antitumor efficacy of PEG‐CCM@APTES‐COF‐1 nanomaterials loading DOX is assessed using a HeLa cell in mice. The as‐prepared 2D COF nanoflakes possess a high specific surface area and ordered structure, which has carved out a novel way for many applications, such as high catalytic ability in electrochemical fields,^[^ ^186^ ^]^ drug delivery carriers, and other biomedical fields.^[^ ^187^, ^188^ ^]^ Moreover, most 2D COFs are made from building blocks with reversible chemical bonds,^[^ ^189^ ^]^ thus making them possible for gradually biodegradable.

## 3 Molecularly Restructured Microenvironments and Biofunctionalities of 2D‐NBPFs

**Scheme 3:**
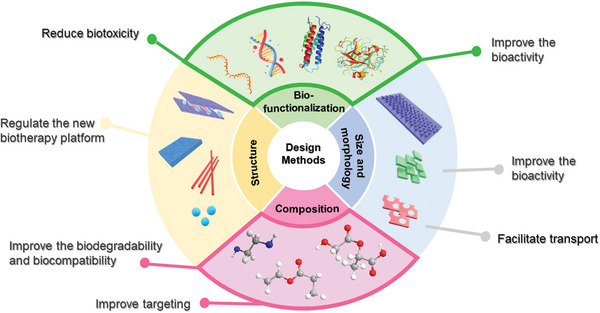
Design methods for engineering advanced therapeutic and diagnostic of 2D‐NBPFs via molecularly restructured microenvironments.

The ultrathin thickness and special structural properties of 2D‐NBPFs provide new prospects for their application in therapy and diagnosis. However, some original 2D‐NBPFs exhibit poor stability and dispersibility, and thus engineering appropriate molecularly restructured microenvironments and biofunctionalities are required to endow pristine 2D‐NBPFs with high stability, dispersity, biocompatibility, and many other physical, chemical, and biological properties. Various designs (**Scheme** **3**) and surface functionalization methods (**Table** **3**) have been applied to improve the properties of 2D‐NBPFs for broader biomedical applications via covalent functionalization, noncovalent modification, and nanoparticle fabrication on 2D‐NBPFs. The design of 2D‐NBPFs can be improved and considered in the following aspects:

1) Surface chemical modification: by changing the material's surface with covalent and noncovalent groups during preparation, the properties of 2D‐NBPFs could be modified and improved. So far, there are two main methods to alter the surface of 2D‐NBPFs: one is covalent interaction, which refers to linking the functional groups (PEG, poly(vinylpyrrolidone) (PVP), poly(*N*‐isopropyl acrylamide) (PNIPAM), methacrylate gelatin, chitosan) to the surface of 2D‐NBPFs^[^ ^55^, ^190^, ^191^ ^]^; the other is noncovalent interaction, which includes hydrogen bonding, *π*–*π* stacking, electrostatic interaction, van der Waals force, etc.^[^ ^43^ ^]^
2) Biofunctionalization: nanomaterials are close to biomolecules in size, so they can be used to create nanoparticle/biological interfaces with biomolecules, namely “biofunctionalization.” Biofunctionalization is also known as the method of surface chemical modification; nevertheless, instead of the above‐mentioned chemical groups, the functional groups also include DNA, protein, soybean phospholipid, activated fibroblast cell membrane, natural killer cell membrane, neutrophil membrane, etc. The biologically functional 2D‐NBPFs have shown promising applications in many fields, including nanotherapies, cellular and tissue images, and tissue regenerative applications.^[^ ^65^, ^125^ ^]^
3) Size and morphology regulation: an in‐depth understanding of the biomedical processes involved in the various emerging therapies can provide estimable information on the size and morphology regulation for 2D‐NBPFs design. The variation of 2D material size and morphology has a great influence on its properties. For example, nanoparticles of ≈20 nm in size are ideal for optimal lymph node‐targeting vaccines.^[^ ^192^ ^]^ Besides, the nanomaterial of a spiny is easier to catch bacteria into with phage.^[^ ^193^ ^]^
4) Structure regulation: by designing 2D‐NBPFs with molecularly programmed microenvironments and definite chemical structures or configurations, the biomedical properties of 2D‐NBPFs are fundamentally improved. In addition, establishing a space‐constrained environment will also change the optical, electrical, magnetic, and biocatalytic properties of 2D‐NBPFs. However, the structure regulatioPn is not universal for all 2D‐NBPFs, for example, single‐element BP.

**Table 3:** Hydrophilicity, biocompatibility, and bioactivity of biomolecules and polymers tailored 2D‐NBPFs and their biofunctionalities

Substrates	Biomolecules & polymers	Biofunctionalities	Potential applications	Refs.
MoS_2_, WS_2_, TiS_2_, BP, MnO_2_, COFs etc.	PEG	Enhanced stability in physiological solutions. Minimized nonspecific biointeractions. Prolonged circulation time. Ability to carry drugs and covert NIR to heat. Versatile postfunctionalization.	Targeted cancer therapy Wound disinfection Tissue engineering	[55, 194, 195, 196, 197, 198, 199, 200, 201, 202, 203]
MoS_2_, Bi_2_S_3_	Chitosan	Ability to carry genes and drugs. Antibacterial effects. Good cell compatibility. Versatile postfunctionalization. pH responsiveness.	Drug and gene delivery Wound disinfection Tissue engineering	[204, 205, 206]
MoSe_2_	PNIPAM	Enhanced stability in physiological solutions. Thermal responsiveness.	Drug delivery Actuator	[207, 208, 209]
Bi_2_S_3_, *h*‐BN, MoS_2_	Arginine‐glycine‐aspartic acid (RGD)	Binding to the integrin of cells, especially the cells overexpressed integrin.	Targeted cancer therapy Biosensor Tissue engineering	[191, 205, 210, 211]
Silicate	Methacrylate gelatin	Cross‐linking upon ultraviolet (UV). Adhesive to tissues. Good cell compatibility. Versatile processability.	Tissue engineering Nanotherapeutics	[212, 213]
MoS_2_, WS_2_, TaS_2_, Ta_2_NiS_5_, MOFs, etc.	DNA	1. Versatile processability. 2. Adsorption of toxins. 2. Binding to specific DNA.	Blood purification DNA sensor	[180, 213, 214, 215, 216]
MnO_2_	Upconversion nanoparticles	Fluorescence signal of Upconversion nanoparticles. pH‐responsive degradability of MnO_2_.	Biosensors	[217]
MnO_2_, Ti_3_C_2_, Ta_4_C_3_	Soybean phospholipid	Enhanced stability in physiological solutions. Minimized nonspecific biointeractions. Prolonged circulation time. Ability to carry drugs and covert NIR to heat. Photoacoustic imaging.	Cancer therapy Bioimaging	[2, 134, 155, 218, 219, 220, 221]
MOFs	PVP	Catalyze H_2_O_2_ reduction	H_2_O_2_ sensor	[222, 223]
LDH	DNA Fluorescein isothiocyanate	Enhanced stability in physiological solutions. Enhanced temporal resolution and sensitivity	Bioimaging Drug and gene delivery	[224]

In summary, 2D‐NBPFs designed to manipulate biological processes carefully, especially in relation to bioadhesive nanointerfaces, specific binding to pathogenic cells, broad‐spectrum pathogen inhibitors, and enzyme‐mimetic 2D‐NBPFs, will allow for more refined applications.

### 3.1 Physiologically‐Stable and Biocompatible 2D‐NBPFs

For biomedical usages, the interaction with biological fluids containing peptides, polysaccharides, proteins, and DNA is inevitable. But the nonspecific interactions are not favored because they will decrease the functionalities of devices and even accelerate immune clearance. Especially for the 2D‐NBPFs, the large surface and strong *π*–*π* and hydrophobic interaction will facilitate the nonspecific biological interactions. Therefore, proper modification with hydrophilic and antifouling polymers is quite necessary, including but not limited to PEG,^[^ ^225^ ^]^ polyglycerol (PG),^[^ ^226^ ^]^ PVP,^[^ ^227^ ^]^ polyaniline (PANI),^[^ ^228^ ^]^ zwitterionic polymers,^[^ ^229^ ^]^ and polysaccharides.^[^ ^230^ ^]^

**Figure 8:**
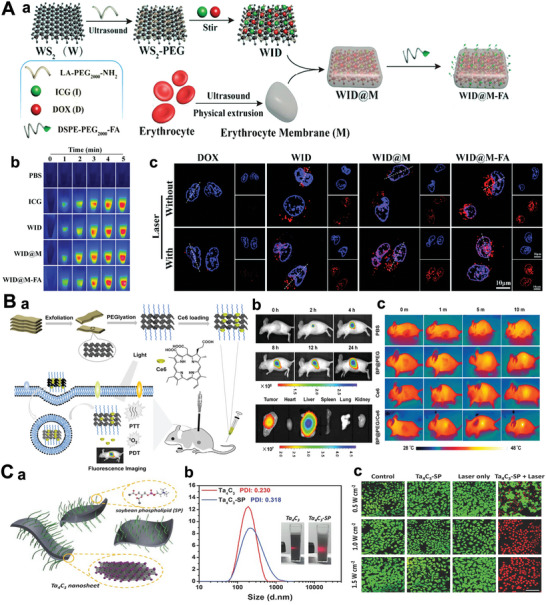
A(a)) Schematic diagram of the synthesis of WID@M‐FA (WS_2_ nanosheets (W), DOX (D), NIR probe indocyanine green (ICG, I)). (b) Photothermal heating images analysis. (c) Confocal laser scanning microscope images of HeLa cells after incubation. Reproduced with permission.^[^ ^8^ ^]^ Copyright 2020, The Royal Society of Chemistry. B(a)) Schematic image of BP@PEG/Ce6 preparation. (b) Time‐lapse fluorescence bioimaging of nude mice. (c) In vivo photothermal effect with 660 nm laser irradiation. Reproduced with permission.^[^ ^194^ ^]^ Copyright 2018, American Chemical Society. C(a)) Schematic representation of a surface modification of Ta_4_C_3_ nanosheets using SP (Ta_4_C_3_‐SP). (b) Dynamic light scattering size distribution profiles of Ta_4_C_3_ and Ta_4_C_3_‐SP nanosheets dispersed in water. (c) Confocal fluorescence imaging of Ta_4_C_3_‐SP induced photothermal ablation after various treatments. Reproduced with permission.^[^ ^156^ ^]^ Copyright 2018, Wiley‐VCH.

Inspired by the previous success of carbon nanotubes and graphene, modification of 2D‐NBPFs by PEG has been studied world‐widely. The WID@M‐FA nanoparticles (NPs) with good physiological stability and biocompatibility were fabricated through adsorbing DOX and ICG (**Figure** **8**A(a)) after surface modification with the erythrocyte membrane (M) and folic acid molecule.^[^ ^8^ ^]^ It showed no obvious toxicity toward HeLa cells (Figure 8A(b,c)). The modification of PEG prolongs the duration time of the 2D nanosheets in the blood, avoids the rapid recognition and elimination of the material by the immune system, and gradually releases the drug in the tumor while promoting tumor imaging. The BP/MoS_2_/TiS_2_ PEG can also be modified by PEG,^[^ ^194^ ^]^ which has been applied to fluorescence and thermal imaging‐guided PTT and PDT (Figure 8B). The reactive oxygen species (ROS) analysis showed no apparent long‐term toxicity of these three types of PEGylated 2D‐NBPFs.^[^ ^169^ ^]^ The PEGylation has also been performed on WS_2_, ReS_2_, MnO_2_, and BP, and these nanosheets showed increased solubility, stability, and decreased toxicity as their counterparts.^[^ ^231^, ^232^, ^233^, ^234^, ^235^, ^236^, ^237^ ^]^ Owing to their high surface area and good photothermal conversion efficiency, they are considered good candidates for drug delivery vehicles and photothermal agents for tumors.

Besides PEG, other hydrophilic polymers have also been investigated for the surface decoration of 2D nanosheets. Polyglycerols (PGs), in terms of linear polymer (lPG), hyperbranched polymer (hPG), and dendrimer (dPG), have been developed with good chemical stability, bioinertness, and high biocompatibility.^[^ ^238^ ^]^ Because of the existence of numerous hydroxyl groups, PG shows excellent water uptake and hence prevents nonspecific protein fouling to surfaces by forming a hydrated layer.^[^ ^239^ ^]^ Another kind of widely studied hydrophilic polymer is the zwitterionic polymers, e.g., poly(sulfobetaine methacrylate) (pSBMA) and poly(carboxybetaine methacrylate) (pCBMA), which contain an equal number of anions and cations on the repeating unit. Zwitterionic polymers can form a hydration layer through the solvation of charged terminal groups, which makes them super antifouling toward the variety of proteins and biological molecules.^[^ ^240^ ^]^ Phospholipid is a major component of cellular membrane; it has a hydrophilic zwitterionic tail and a hydrophobic fatty acid tail. For the 2D nanosheets, phospholipids adhere to the surface readily via the hydrophobic interaction of the basal plane. Recently, Lin et al. attached soybean phospholipids (SP) onto Ta_4_C_3_ 2D nanosheets, and they noticed that SP‐Ta_4_C_3_ had better dispersity and stability in water than neat Ta_4_C_3_ (Figure 8C).^[^ ^156^ ^]^ Similarly, the SP modified MnOx/Ti_2_C_3_ composite nanosheet showed better activity than bare MnO*_x_*/Ti_2_C_3_ for MRI imaging and photothermal therapy due to the increased stability in water.^[^ ^221^ ^]^ Natural killer cells, as a kind of innate immune effector cells, have attracted extensive attention in the field of tumor immunotherapy. In antitumor immunotherapy, natural killer cells can induce macrophages to polarize toward inflammatory M1 type for tumor targeting via proteins in natural killer cell membranes, such as RANKL or DNAM‐1.^[^ ^241^ ^]^ Therefore, the 2D‐NBPFs modified by natural killer all membranes may have better tumor treatment effects.

The surface modification of 2D‐NBPFs is an efficient method to enhance physiological stability and biocompatibility, which has been investigated in numerous scientific papers. Some of the research proves that the coupling of molecules on the surface of 2D‐NBPFs can effectively improve biocompatibility, which is due to the stability of the cell membrane being affected by changing the surface charge and the activity of chemical groups. In addition, the addition of specific molecules can also enhance the passive and active uptake of 2D nanosheets, reduce systemic toxicity in vivo, and enable high‐precision therapy and/or diagnosis.

Polysaccharides play an important role in animal growth as a power resource and promoters for cell proliferation, tissue building, and immune‐modulating. It has been pointed out that polysaccharide‐cell interactions are essential for cell receptor engagement and recognition in extra‐cellular‐matrix. Chitosan is a positively charged polysaccharide with numerous amino groups, and it can be loaded onto 2D‐NBPF surfaces via either electrostatic interaction or covalent linkage. It has been noticed that modification of MoS_2_ by chitosan shows improved compatibility toward red blood cells, human epithelial carcinoma cell lines, and epithelial‐like cell lines.^[^ ^242^ ^]^ Due to the cationic nature of chitosan in an aqueous solution, chitosan shows reversible interaction with DNA, and it can protect DNA from degradation as a gene delivery vehicle.^[^ ^243^ ^]^ Besides the delivery nanovehicle, chitosan has also been processed into fibers, films, and porous scaffolds with 2D‐NBPFs as wound dressing and tissue‐rebuilding materials. 2D‐NBPFs could generate porous structure in chitosan scaffold to extend contacting sites with cells and tissues without decreasing the mechanical property.^[^ ^244^, ^245^, ^246^ ^]^ There are also many other kinds of biocompatible 2D‐NBPFs that have been designed with hyaluronic acid (HA),^[^ ^247^ ^]^ cellulose,^[^ ^248^ ^]^ and others, and all of them give the similar conclusion that after the functionalization of polysaccharides, the biocompatibility of 2D‐NBPFs can be significantly increased.

### 3.2 Bioadhesiveness and Internalization at Nanointerfaces

By changing the surface functional group of 2D‐NBPFs, the materials can realize biological drug loading and exhibit excellent biocompatibility.^[^ ^249^ ^]^ There are three functional molecules, including heparin/chitosan/mussel, as effective biological modification of 2D‐NBPFs, could produce adhesiveness and internalization at nano‐interface. Due to the anticoagulant effect of heparin‐based polymers, many heparin‐based polymers have been developed and achieved biological activities comparable to natural heparins, such as preventing coagulation and thrombosis and promoting endothelial cell growth.^[^ ^250^ ^]^ Among the polysaccharides for drug delivery, chitosan has been widely used due to its bioadhesive and internalized properties. This is due to its high molecular weight and degree of deacetylation, which forms a gel at acidic pH. In addition, it mostly exhibits cationic properties, which enables it to bind tightly to animal cells.^[^ ^251^ ^]^ The mussel also is adhesive to virtually all types of surfaces, and it is also found that the dopamine‐coated surface can promote cell adhesion and internalization due to the affinity to the cell surface.^[^ ^252^, ^253^ ^]^ For 2D‐NBPFs, mussel‐inspired polydopamine (PDA) coating not only increases the water solubility and stability but also enhances cell interaction. For cell adhesive nanosystems, RGD, one of the most commonly used peptide motifs, which recognizes the integrins on the cell surface. Introducing RGD onto 2D nanosheet surfaces has been achieved by both covalent and noncovalent approaches.^[^ ^254^ ^]^ For example, the RGD and Bi_2_S_3_ nanosheet together as a multifunctional targeted theranostic platform was used for antitumor therapies.^[^ ^255^ ^]^ Through the RGD‐integrin targeting, the Bi_2_S_3_‐RGD nanocomposites showed enhanced cell uptake and tumor accumulation and allowed the imaging of tumors and photothermal therapy. Besides targeted cancer therapy, the RGD functionalized 2D nanosheets have also been used for tissue engineering and in vivo biosensing. However, mussel‐based adhesives easily lose their adhesion due to the easy oxidation of the catechol groups and 3,4‐dihydroxy phenylalanine on their backbones, which limits their applications in biomedicines.

### 3.3 Specific Binding to Pathogen

Many severe diseases are caused by viral infections, and newly emerged viruses are major threats. Blocking virus/cell interactions to combat viral infections has gradually become an effective strategy. However, polymeric multivalent inhibitors have too small surfaces compared to viruses (about 50–300 nm); therefore, the contact area is rather limited. 2D‐NBPFs are a more promising scaffold for viral interaction and inhibition, providing a larger contact and more multivalent ligand representation. Since most viruses have positively charged surfaces, the negatively charged 2D‐NBPFs, especially the sulfated nanosheets, have demonstrated strong virus binding.^[^ ^256^ ^]^ Besides, 2D‐NBPFs could bind with pathogenic bacteria by surface functionalization. There are reports that glucan multivalent interactions for the stripping and functionalization of 2D TMDs‐based NBPFs in an aqueous solution. During the liquid exfoliation process, the multivalent hydrogen bonds of dextran and bulk TMDs can effectively generate TMD monolayers that bind to pathogenic bacteria multivalently.

2D‐NBPFs have a multifunctional group and porous structure, which is beneficial for loading monoclonal antibodies or small interfering ribonucleic acid for immune checkpoint blocking therapy.

Currently, immune checkpoint blocking therapy has become a promising therapeutic strategy, which activates the immune system to inhibit the occurrence and metastasis of tumors. Programmed death ligand 1 (PD‐L1) is considered a key regulator of tumor‐related immunosuppression and tumor progression, and it has been used as a therapeutic target in the clinical treatment of solid tumors.^[^ ^257^ ^]^ 2D‐NBFFs, such as MOF and COF could be used to load, protect, and deliver small interfering ribonucleic acid, which avoids the degradation of small interfering ribonucleic acid by enzymatic. Therefore, 2D‐NBPFs have broad application prospects in improving the efficacy and efficiency of gene therapy.

### 3.4 Broad‐Spectrum Pathogen Inhibitors

In addition to specific binding to the pathogens, the induction of oxidative stress is another generally accepted antipathogen mechanism of 2D‐NBPFs.^[^ ^258^ ^]^ 2D‐NBPFs‐induced oxidative stress can be divided into two types: ROS dependencies and ROS nondependent methods. The difference between the two is that the former is related to the production of ROS and the accumulation of damage cells, and the latter is usually related to the consuming cell antioxidant consumed by the transfer of charge. Both can lead to lipid peroxidation, which destroys the membrane structure of the bacteria. It can also cause dysfunction of biomolecular dysfunction of biomolecularity such as protein and DNA/RNA, which eventually causes bacterial cells to die. In addition, via a synergistic effect between physical damage and chemical oxidation, the pathogen can be killed quickly and constantly. For example, the “blade‐like” edge of MoS_2_ nanosheets cut through the cell membrane/extracts lipids. Then, ROS would be produced by the highly effective electron transfer behavior of Mo. Finally, efficient antibacterial effects can be achieved through the synergistic action.^[^ ^259^ ^]^

The efficacy of 2D‐NBPFs depends on several factors, including thickness, lateral size, structure, and surface functional groups. For example, Moaty et al. prepared a bimetallic loaded LDH and discovered its durable broad‐spectrum antimicrobial activities against Gram‐negative and Gram‐positive bacteria, superbugs, and fungi.^[^ ^260^ ^]^ In addition, 2D‐NBPFs have a large surface area and rich functional group, which can be loaded with drugs for antibacterial applications.

### 3.5 Stimuli‐Responsive Nano‐Bio‐Systems

Living cells can adapt to the local environments through the biomacromolecules that alter properties, such as shape, stiffness, and hydrophilicity, in response to external stimuli. For biomedical materials, making them “smart” can give better biological performance as it recognizes the stimuli and allows on‐demand control of their bioactivities.^[^ ^261^ ^]^ Temperature responsiveness, thermal responsiveness, are more studied strategies for a stimuli‐responsive system. Polymeric material, it can undergo property changes with increasing temperatures due to the increased chain mobility. In addition, to the effects of entropy and enthalpy, some polymers show temperature‐dependent solubility in water. One of the most studied responsive polymers is poly(*N*‐isopropyl acrylamide), which undergoes a sharp coil‐globule transition in the water at around 35 °C and a change from hydrophilic state ta o hydrophobic state with increased temperature. In addition, copolymerization of poly(*N*‐isopropyl acrylamide) with other polymers in a wide range of transition temperatures has also been developed.

**Figure 9:**
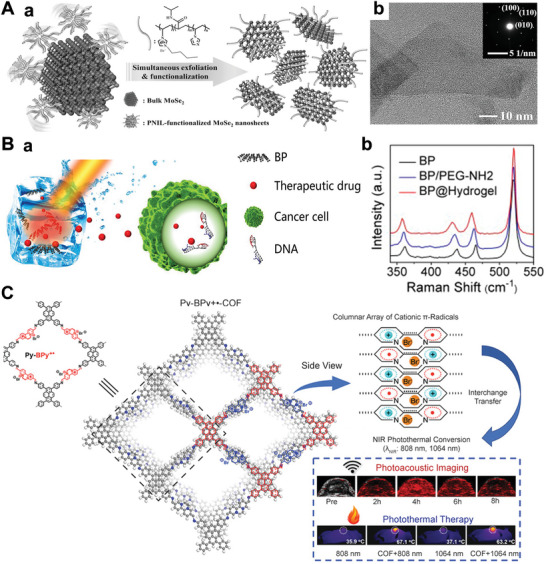
A(a)) Scheme for preparation of PNIL‐MoSe_2_ hydrogels. (b) HRTEM images of the MoSe_2_ nanosheets. Reproduced with permission.^[^ ^262^ ^]^ Copyright 2016, Wiley‐VCH. B(a)) Schematic illustration of the working principle of BP@Hydrogel. (b) Raman spectra image of BP, BP/PEG‐NH_2_, and BP@Hydrogel. Reproduced with permission.^[^ ^263^ ^]^ Copyright 2018, National Academy of Sciences. C) The PEG functionalized Py‐BPy+•−COF (Py‐BPy+•−COF/PEG) exhibits good water‐soluble and photothermal therapies as well as PTT in mice. Reproduced with permission.^[^ ^114^ ^]^ Copyright 2019, American Chemical Society.

Light can offer the possibility of remote control of biofunctionalities for the target area. A large variety of nanomaterials that respond to UV, visible, and NIR irradiation have been developed to achieve on‐demand drug delivery. NIR in the range of 700–900 nm is much more preferred than UV and visible light for biomedical applications since it is more penetrative and shows less toxicity. Due to the unique structure and tunable bandgap, 2D‐NBPFs can generate heat upon NIR irradiation, which has been utilized for cancer therapy and diagnosis. Combining photothermal efficiency with 2D‐NBPFs, the NIR‐responsive MoSe_2_ nanosheets were developed (**Figure** **9**A(a,b)), and then it was polymerized with NIPAM to form a dual stimuli‐responsive (poly(*N*‐isopropyl acrylamide‐*co*‐IL), PNIL)‐MoSe_2_ hydrogel, of which the shrink and swell could be controlled by NIR irradiation, leading to the boosted release of DOX under the laser.^[^ ^262^ ^]^ Similarly, agarose‐modified BP nanosheets could be used as a NIR‐responsive hydrogel delivery platform for cancer therapy.^[^ ^263^ ^]^ Upon NIR irradiation, the heat was generated by BP nanosheet to soften agarose hydrogel, which would then trigger DOX release and significantly inhibit tumor growth due to combined photothermal therapy and chemotherapy. Moreover, the combination of 2D‐NBPFs and responsive polymers also led to the development of NIR actuators, of which the generation of heat could trigger the movement of devices^[^ ^263^ ^]^ (Figure 9B(a,b)). Besides the good biocompatibility, 2D‐NBPFs NIR actuator showed great potential for remote control of biomedical robots for precision surgery.

The high conjugation of 2D COFs endows them with unique photothermal responsive properties. For example, a 2D COF‐containing radical cation (Py‐BPy+•−COF)^[^ ^114^ ^]^ was developed via an in situ reaction method that leads to stabilization and delocalization (Figure 9C). The extinction coefficients of the PEG‐functionalized aqueous dispersant Py‐BPy+•−COF/PEG at 808 and 1064 nm are 16.6 and 15.9 L g^−1^ cm^−1^, respectively, which exhibits significant near‐infrared absorption properties. Therefore, COF‐based photothermal agents can efficiently stimulate responsiveness and serve as ideal drug carriers.

In addition, tumor microenvironment response is also an important stimuli‐responsive nano‐bio‐systems, including pH response and reduction response.^[^ ^264^ ^]^ The high glycolysis rate at the tumor resulted in the accumulation of acid metabolites, and the pH value of the tumor extracellular environment was reduced to ≈6.5. The slightly acidic nature of tumors is often used as a stimulus for responsive drug delivery systems. Based on pH responsiveness, the general carrier type is ionizable groups‐containing polymers, of which conformation and solubility will change under different pH values. And other 2D materials contain acid‐sensitive fracture groups (such as hydrazone, imine, acylhydrazone, acetal/copper bonds, etc.), which undergo responsive fracture at a specific pH value. For example, Trabolsi's team designed a multifunctional magnetic COF, named TAB‐DFP‐nCOF, which was successfully applied as MRI, chemotherapy, and hyperthermia agents. The structure of TAB‐DFP‐nCOF was destroyed and degraded through the natural pathway of endocytosis, eventually releasing the drug under the acidic media due to the imine bond break.^[^ ^265^ ^]^ In addition, the glutathione (GSH) content in tumor cells is much higher than that in normal cells.^[^ ^266^ ^]^ Disulfide bond is the most commonly used chemical bond in the design of GSH‐responsive drug delivery system. By a inserting double bond into the structure of the nanodrug delivery system, GSH‐responsive controlled drug release can be achieved. For instance, Cu (II)‐based 2D is F as a unique chemodynamic therapy (CDT) nanoagents for GSH‐triggered and H_2_O_2_‐augmented toward remarkable tumor suppression. Subsequently, abundant hydroxyl radicals generation was achieved via Fenton‐like catalytic reaction leading to cellular apoptosis and tumor inhibition.^[^ ^267^ ^]^

### 3.6 Enzyme‐Mimetic 2D‐NBPFs

**Figure 10:**
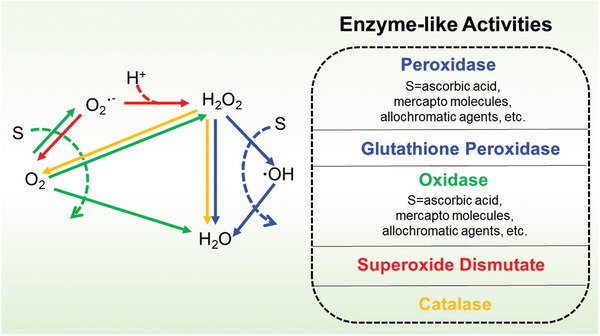
Schematic representation of typical mimicking enzyme activity. Reproduced with permission.^[^ ^276^ ^]^ Copyright 2022, The Royal Society of Chemistry.

Because of the high specific surface area, 2D‐NBPF has been recognized as a versatile platform to load bioactive nanoparticles as artificial enzymes to catalyze bioreactions. Moreover, the 2D‐NBPF characteristics further make them with new biomedical performances, which are expected to complete biological applications that enzymes cannot. For example, 2D‐NBPFs as emerging catalysts can achieve the mimics of various enzymes, such as oxidase (OXD), peroxidase (POD), superoxide dismutase (SOD), catalase (CAT), glutathione peroxidase (GPx), and hydrolase (HYL) (**Figure** **10**).

**Figure 11:**
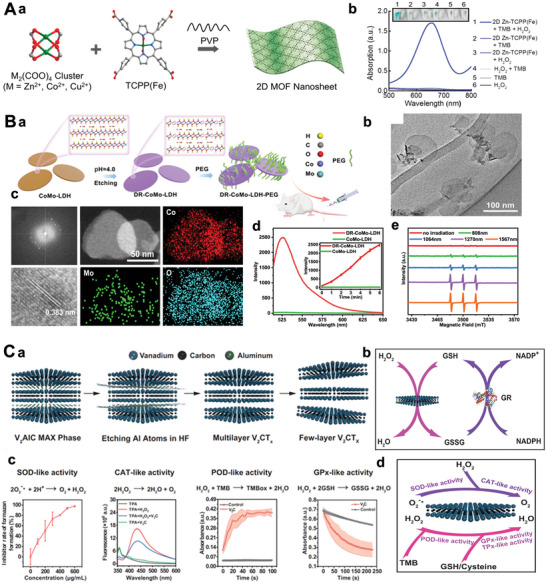
A(a)) The schematic image of synthetic 2D MOF. (b) UV–vis spectra that prove the good performance of the Zn‐TCPP(Fe) enzyme. Reproduced with permission.^[^ ^271^ ^]^ Copyright 2019, American Chemical Society. B(a)) Schematic diagram of the CoMo‐LDH. (b) TEM characterization (c) HRTEM image and EDX mapping of CoMo‐LDH. (d) The fluorescence intensity of the singlet oxygen sensor is green in the presence of the CoMo‐LDH and DR‐CoMo‐LDH nanosheets. (e) ESR spectra of singlet oxygen (^1^O_2_) for the DR‐CoMo‐LDH nanosheets. DR (defect‐rich). Reproduced with permission.^[^ ^103^ ^]^ Copyright 2022, Springer Nature. C(a)) Schematic image of fabricating V_2_C MXene. (b) The image of enzyme‐mimicking activities of V_2_C nanoenzyme. (c) SOD/CAT/POD/GPx‐like activity of V_2_C nanoenzyme. (d) The image of GPx‐like enzyme activities and GSH recycling of V_2_C MXenzyme by glutathione reductase (GR). Reproduced with permission.^[^ ^277^ ^]^ Copyright 2021, Springer Nature.

Many 2D MOFs have been reported to show POD‐mimicking properties.^[^ ^268^, ^269^, ^270^ ^]^ For instance, 2D MOF materials Zn‐TCCP(Fe/Cu/Co)^[^ ^271^ ^]^ have POD‐mimicking properties and the POD performance of 2D MOFs depend on its active center (**Figure** **11**A(a,b)). The active center of metal coordinated TCCP compound is similar to natural POD enzyme. And it exhibits higher activity than original bulk materials, which is due to a larger contact area and lesser diffusion resistance of 2D Zn‐TCPP(Fe).

LDH, as a representative ionic layered material with distinct coordination structures, was usually used in various medical diagnosis and treatment fields. If LDH contains more defective structures will enhance the performance of the mimetic application. The hydrothermally synthesized CoMo‐LDH and NiMo‐LDH are etched by acid cleaning to create defect‐rich nanosheets (Figure 11B(a–c)). Especially, CoMo‐LDH with abundant defect structure exhibits higher ROS performance than that of the originally untreated CoMo‐LDH under a NIR irradiation (Figure 11B(d,e)), which could effectively induce cancer cell apoptosis in vitro and in vitro. This manifests that LDHs‐based nanosheets are very prospective materials for designing multifunctional enzyme‐mimetic 2D‐NBPFs.

TMDs also have been reported POD mimic‐enzyme properties, including MoS_2_, WS_2_, and VS_2_.^[^ ^272^, ^273^, ^274^ ^]^ The simulated enzyme properties of TMDs are related to the Fermi level. For example, MoS_2_ as a TMDs with a suitable Fermi level can induce electron transfer, which promotes the ROS produced from H_2_O_2_ and the TMB oxidated. It is because the defect sites are led to the high enzymatic activity of TMDs. The principle is that the energy level is different between the HOMO of TMB and the LOMO of H_2_O_2_; the electron transfer is prevented in the Fenton reaction^[^ ^275^ ^]^ when the Fermi level is suitable; therefore, the defect site can enhance the enzymatic activity of TMD.

However, an artificial enzyme with a single function cannot mimic the natural antioxidant system in cells to fight oxidative stress. 2D vanadium carbide (V_2_C) MXene nanoenzyme as a multifunction nanoreactor simulates the complicated intracellular enzyme participated ROS defense system. It can mimic six natural enzymes, such as SOD, CAT, POD, GPx, and halogen‐peroxidase (HPO). Notably, 2D V_2_C nanozymes have promising in vivo therapeutic effects for relieving ROS‐induced damage and re‐establishing redox homeostasis without interfering with antioxidant status, as demonstrated in animal models of both inflammation and neurodegeneration. 2D V_2_C nanoenzyme reconstructs the redox homeostasis without obstructing the antioxidant status and relieves ROS‐caused injury with good in vivo therapeutic effects, as proved by disease models in animal models.

Besides, the enzyme‐mimetic property of 2D‐NBPFs depends on pH value. At neutral pH, some of the 2D‐NBPFs exhibit CAT‐like activities and can play a role in scavenging ROS. Under acidic conditions, these enzymatic 2D‐NBPFs exhibit POD‐like activities to produce ROS.

## 4 Therapeutic and Diagnostic Applications of 2D‐NBPFs

**Scheme 4:**
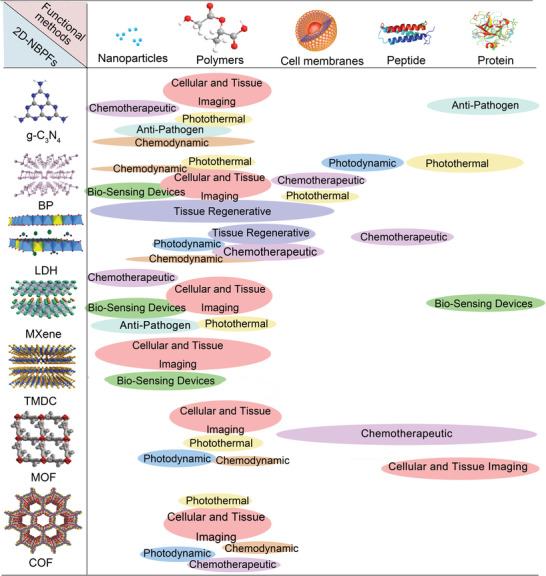
Summary of the key functional properties of various 2D engineering advanced therapeutic and diagnostic nano‐bio‐platforms and applications in advanced therapy design.

2D‐NBPFs have been applicated to various fields, including biomedical diagnostics and therapeutics aspects, due to their prominent physical, chemical, electronic, optical, and magnetic properties. However, it remains a severe challenge how to integrate their unique properties into the function of a therapeutic 2D‐NBPFs system. Hence, it is necessary to comprehend the mechanisms of various biological processes and how to manipulate the properties of bioderived compounds to construct sound systems. We established a framework to predict the performance and function of composite 2D‐NBPFs composed of 2D materials (**Scheme** **4**), which will facilitate easier application. In the following sections, the biomedical applications of 2D‐NBPFs and compounds are discussed in depth.

### 4.1 Chemotherapy

**Figure 12:**
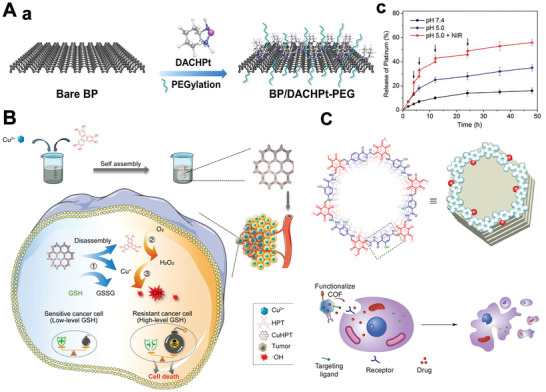
A(a)) Synthetic method of BP/DACHPt‐PEG. (b) Release kinetics of BP/DACHPt at different pH levels (with/without NIR irradiation). Reproduced with permission.^[^ ^283^ ^]^ Copyright 2019, Elsevier. B) Schematic image of the anticancer process of copper/catechol‐based metal–organic framework (CuHPT). Reproduced with permission.^[^ ^285^ ^]^ Copyright 2022, American Chemical Society. C) A *β*‐ketamine based 2D COFs TpASH promotes targeted delivery of 5‐fluorouracil in tumor cells. Reproduced with permission.^[^ ^115^ ^]^ Copyright 2017, American Chemical Society.

With every atom exposed on the surface, thin‐layered 2D‐NBPFs show abundant binding sites for molecule uptakes, which has promoted the development of 2D‐NBPFs‐based drug/gene delivery vehicles.^[^ ^278^, ^279^ ^]^ Most existing nanocarriers result in poor drug loading and/or fast drug release due to simple physical or chemical adsorption on the surface of the nanocarrier.^[^ ^280^, ^281^, ^282^ ^]^ Surface modification is also a significant method to enhance the biosafety of 2D‐NBPFs. The active components of anticancer drugs (1,2‐cyclohexaneohexanee)platinum(II), DACHPt) were used to synergy with BP nanosheets to form complex BP/DACHPt and improve their stability,^[^ ^283^ ^]^ which was fabricated for chemo‐photothermal synergistic tumor therapy (**Figure** **12**A(a)). The BP/DACHPt‐PEG nanosheets were successfully prepared by combining DACHPt with a modified BP. As shown in Figure 12A(b), the DACHPt release kinetics of BP/DACHPt‐PEG were investigated with two kinds of pH under 808 nm NIR laser exposure or no laser exposure. DACHPt was released continuously and delayed at different pH conditions, which is due to the strength change of the synergy bonds between DACHPt and BP with pH.^[^ ^284^ ^]^ The NIR laser irradiation could cause momentary burst release, thus achieving a highly effective antitumor effect. Furthermore, low toxicity for the major organs and biodegradability, makes BP appropriate for chemotherapy applications in vivo.

Chemotherapy resistance is an essential issue in tumor therapy. Due to the complexity of chemoresistance mechanisms, targeted drugs and molecular inhibitors usually cannot eliminate drug‐resistant tumor cells. Here, based on the characterization of high‐level but steady‐state biochemical fragility of intracellular homeostasis environment in drug‐fast tumor cells, the author engineered a nanosized CuHPT, which can perturb homeostasis and occur oxidative stress (Figure 12B).^[^ ^285^ ^]^ It enhances intracellular ROS production through auto‐oxidation and GSH‐depleting Fenton reactions. In addition, the targeting of the material is also critical, which can minimize the material's toxicity to normal cells. Due to the surface modifiability of COF‐based 2D‐NBPFs, the targeted delivery of drugs can be achieved by functionalizing them with folic acid. For example, Banerjee et al. reported a *β*‐ketamine‐based 2D COFs (TpASH, Figure 12C) by a modification method with postsynthetic,^[^ ^115^ ^]^ which were surface‐functionalized with folate. The unencapsulated drug in the supernatant was evaluated by UV visible spectroscopy, and it was found that the drug loading efficiency of TpASH was 12%. 5‐Fluorouracil was continuously released for 3 d at pH 5 in tumor cell lysosomes (the release rate was 74%). Folate receptor‐positive human triple‐negative breast cancer cells (MDA‐MB‐231) selectively internalize 5‐fluorouracil through folate receptor‐mediated endocytosis to induce programmed cell death.

### 4.2 Chemodynamic Therapy

**Figure 13:**
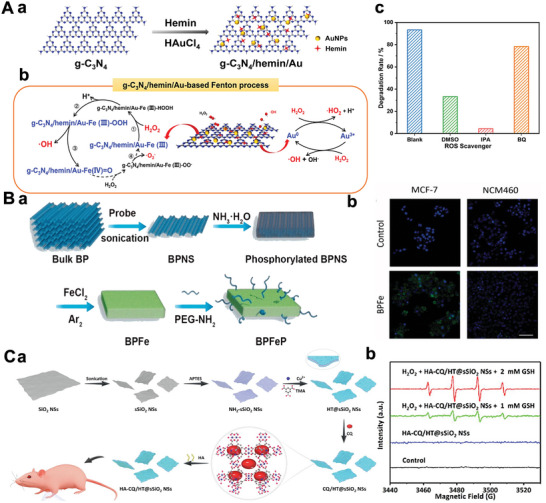
A(a)) Schematic image of preparation of g‐C_3_N_4_/hemin/Au. (b) Mechanism of Fenton catalytic process. (c) The histogram of TMB catalyzed via g‐C_3_N_4_/hemin/Au. Reproduced with permission.^[^ ^288^ ^]^ Copyright 2020, Elsevier. B(a)) Schematic illustration preparation of BPFe. (b) Confocal images of fluorescence intensity. Reproduced with permission.^[^ ^290^ ^]^ Copyright 2022, Elsevier. C(a)) Schematic illustration preparation of sandwich‐like 2D nanosheets. (b) ESR spectra of HA‐CQ/HT@sSiO_2_ nanosheets under the different solvents. Reproduced with permission.^[^ ^292^ ^]^ Copyright 2021, Elsevier.

Chemodynamic therapy (CDT) is used in tumor treatment by breaking down H_2_O_2_ to produce abundant ROS in the tumor microenvironment to damage tumor cells.^[^ ^54^ ^]^ However, insufficient endogenous H_2_O_2_ usually reduces the CDT effect because intracellular up‐regulated reductants, would neutralize ROS.^[^ ^286^ ^]^ An effective therapeutic modality is that metal ions with high oxidation states were used as Fenton‐like reagents for scavenging reductants. 2D materials containing transition metal or precious metal exhibited remarkable antitumor effects via typical Fenton reactions to generate multiple free radicals.

g‐C_3_N_4_‐based biomimetic nanocatalyst as a novel 2D‐NBPF has been widely explored in CDT.^[^ ^287^ ^]^ As shown in **Figure** **13**A(a), the Au‐supported g‐C_3_N_4_/hemin nanohybrid is prepared to enhance CDT effectiveness. The raised Fenton catalytic performance of the g‐C_3_N_4_ based nanohybrid can be attributed to the high affinity between H_2_O_2_ and nanohybrid.^[^ ^288^ ^]^ Moreover, it is also because the direct conversion of Fe(III) to Fe(IV) does not form inert Fe(OH)x (Figure 13A(b)), which leads to a higher metal content with high oxidation states. As a biomimetic nanocatalyst, the 2D nanomaterial g‐C_3_N_4_/hemin/Au provides outstanding CDT property in the presence of H_2_O_2_ at both acidic and neutral pH (Figure 13A(c)).

The characteristic of BP‐based 2D‐NBPFs facilitates favorable cationic loading, which expanded its application in the field of biomedicine.^[^ ^289^ ^]^ For example, an iron‐mineralized BP nanosheet (BPFe) was designed to deliver and release Fe ions on‐demand to tumor sites (Figure 13B(a)), realizing CDT cancer therapy.^[^ ^290^ ^]^ Due to the rapid release of the loaded Fe ions in the acidic environment of the tumor, BPFe exhibits a better Fenton catalytic effect than the usual iron oxide‐based nanocatalysts (Figure 13B(b)).

LDH‐based nanocatalysts are composed of abundant positive charge and hydroxyl group on the surface, which benefit from loading metal ions.^[^ ^291^ ^]^ Besides, LDH with large amounts of defect structures possess higher Fenton activity than traditional LDH‐based materials. For example, the defect‐rich CoMo‐LDH nanosheets exhibit higher ROS generation activity (≈97 times) than the pristine CoMo‐LDH nanosheets.^[^ ^103^ ^]^

MOF can be subtly developed as a favorable candidate for controllable CDT via introducing the Fenton‐type transition metal node. Compared with 3D MOF, 2D‐MOF nanosheets are more promising in the preparation of CDT nanoplatforms because of their typical planar topology, ultrathin thickness, and large specific surface area. For instance, Lin's group prepared a HA‐CQ/HT@sSiO_2_ nanosheets (Figure 13C(a)), which could effectively accumulate at the tumor site into tumor cells benefiting from unique sheet‐like morphology.^[^ ^292^ ^]^ And it can be used to effectively activate CDTS triggered by autophagy inhibition (Figure 13C(b)).

Since high levels of GSH in cancer cells reduce the expected efficacy of ROS clearance, many studies have combined ROS therapy with GSH depletion strategies to improve the efficacy of the original therapy. Ferrous, copper, manganese and other metals in 2D‐NBPFs can form valence cycles between GSH reduction reaction and Fenton/Fenton‐like reaction, continuously inducing GSH consumption and hydroxyl radical production, eventually enhancing the efficacy of CDT.

### 4.3 Photothermal Therapies

PTT is a significant strategy to combat tumors, which utilizes a light‐absorbent material to generate heat light irradiation conditions, thus leading to efficient ablation of the cancer cells.^[^ ^293^ ^]^ In terms of light‐absorbing agents, many results revealed that 2D‐NBPFs with atomic thickness exhibit high photothermal conversion efficiency at the therapeutic window (700–900 nm). Besides, 2D‐NBPFs can accumulate in tumors due to the high permeability and retention effect of tumor cells. After infrared radiation, 2D‐NBPFs will generate a lot of heat, and the defects of tumor tissue structure as well as the heat dissipation slow, leading to the killing or gradual apoptosis of cancer cells. However, PTT has a tendency to burn the skin and needs to overcome heat shock proteins.

**Figure 14:**
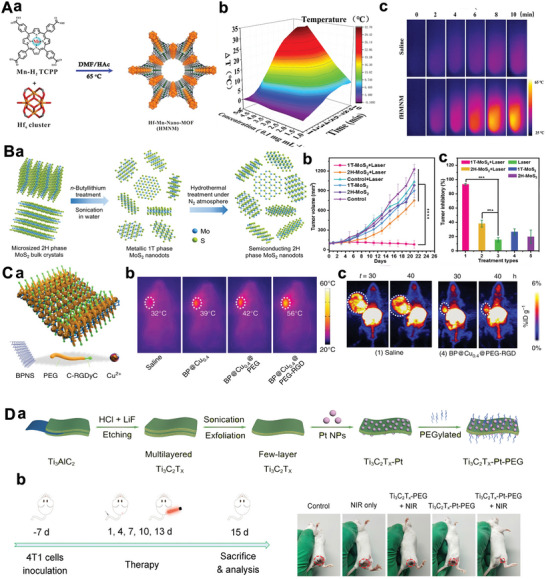
A(a)) Schematic image of FA‐Hf‐Mn‐metal–organic framework (HMNM) was synthesized. (b) Different temperature image with different concentrations of HMNM. (c) Infrared thermal images of HMNM aqueous dispersions. Reproduced with permission.^[^ ^111^ ^]^ Copyright 2020, Elsevier. B(a)) Schematic diagram of the process for the synthesis of metallic 2H‐phase MoS_2_. (b) Tumor growth curve over time. (c) Tumor inhibition rate under different conditions. Reproduced with permission.^[^ ^295^ ^]^ Copyright 2020, Wiley‐VCH. C(a)) Structure model of the structure of BP@Cu@PEG‐RGD. (b) Infrared thermal images of tumor‐bearing mice under 808 nm laser irradiation. (c) MIP PET images of B16F10 tumor‐bearing mice. Reproduced with permission.^[^ ^296^ ^]^ Copyright 2020, Springer Nature. D(a)) Infrared thermographic images of Ti_3_C_2_T*_x_*‐Pt‐PEG composite nanosheets as the PTT agents for 4T1 cell ablation. (b) Images of the mice 15 days after various treatments. Reproduced with permission.^[^ ^18^ ^]^ Copyright 2022, American Chemical Society.

The fast development of 2D‐NBPFs offers a new possibility for designing PTT agents with enhanced photothermal conversion to achieve higher ablation of cancer cells. Zr element‐modified MOFs have exhibited enormous potential in drug delivery because of their tunable sizes/shapes, uniform pore size distribution, controllable morphology, and intrinsic biodegradability.^[^ ^294^ ^]^ In addition, folic acid functionalized hafnium, and manganese‐based MOF, showing great clinical application potential for tumor treatment by PTT (**Figure** **14**A(a–c)).

TMD is commonly used in photothermal antitumor therapy, especially MoS_2_, which has been proven to be appealing nanoagents for PTT.^[^ ^295^ ^]^ Crystal structure of MoS_2_ plays a key role in photoacoustic imaging and photothermal properties of TMD nanomaterials. For instance, the metallic 1T‐MoS_2_ can give stronger photoacoustic imaging signals as compared to signals of 2H‐MoS_2_ in the NIR window (Figure 14B(a–c)). In addition, the PVP modified 1T‐MoS_2_ can be used as a highly efficient agent for photoacoustic imaging guided PTT to effectively ablate cancer cells under 1064 nm laser irradiation.

BP is also one promising nanoagent in PTT due to its extremely high photothermal efficiency.^[^ ^194^ ^]^ However, BP easily degrades and loses its crystallizability under the presence of light, oxygen, and water, which greatly limits its application. A surface modification strategy can improve its stability and expand its application range. As shown in Figure 14C(a–c), the BP of Cu^2+^‐capturing can enhance photothermal stability. The incorporation of Cu^2+^ into BP@Cu nanostructures further enables hemodynamic therapy enhanced PTT.

MXenes have attracted widely used in PTT treatment because of their intrinsic physical and chemical properties. Ta_4_C_3_ with a redox reaction modified can guide the multimodal imaging and photothermal tumor ablation (Figure 14D(a)). As shown in Figure 14D(b), tumors in the group treated with Ti_3_C_2_T*_x_*‐Pt‐PEG + 1064 nm disappeared. These indicated PTT of MnO*_x_*/Ta_4_C_3_–SP nanosheets showing high power‐dependent. Although various MXene‐based nanomaterials have been researched to be useful for PTT, current studies still lack exploration on whether/how MXenes‐based nanomaterials are cleared from the body and their possible long‐term influence on living organisms.

### 4.4 Photodynamic Therapies

**Figure 15:**
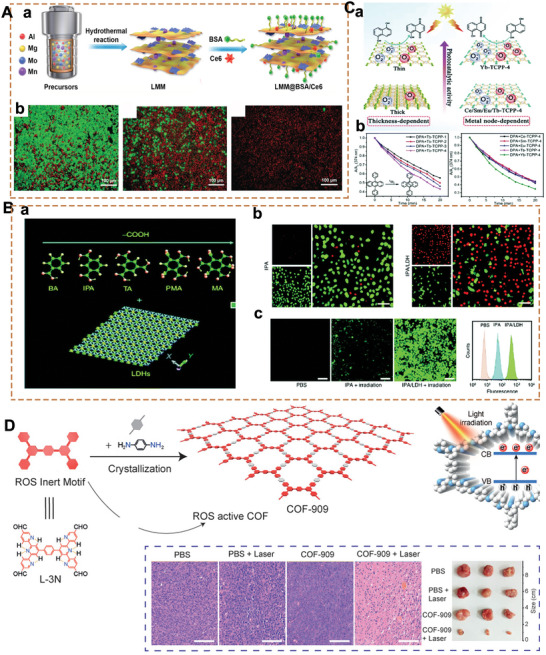
A(a)) Schematic image of LMM@BSA/Ce6. (b) The calcein‐AM/PI dyeing image of HT29 cells. Reproduced with permission.^[^ ^297^ ^]^ Copyright 2021, Springer Nature. B(a)) Illustration image of 2D LDH‐based nanohybrids for producing ^1^O_2_. (b) Calcein‐AM/PI staining analysis of cells cultivated with isophthalic acid (left) and isophthalic acid/LDH (right) following irradiation. (c) The Left is confocal images of intracellular ROS, and the right is flow cytometry measurements. Reproduced with permission.^[^ ^298^ ^]^ Copyright 2018, Springer Nature. C(a)) Schematic image of the fabrication of 2D Ln‐TCPP nanomaterials. (b) Changes in absorbance of diphenylamine in the existence of different thicknesses of 2D Tb‐TCPP nanomaterials (left) and Ln‐TCPP‐4 nanosheets (right). Reproduced with permission.^[^ ^112^ ^]^ Copyright 2020, Wiley‐VCH. D) Schematic image of COF‐909 and PDT efficiency. Reproduced with permission.^[^ ^184^ ^]^ Copyright 2019, Wiley‐VCH.

PDT is another form of cancer phototherapy, which utilize a photosensitizer to produce ROS by light to induce tumor cell apoptosis. There is no process of heat conduction in PDT, so the healthy cells are not subject to heat transmission, which achieves better‐targeted killing of tumor cells. At present, PDT still has shortcomings, such as insufficient ROS generation, insufficient oxygen supply, poor light penetration, and nonspecific biodistribution of photosensitizers. BP‐based 2D‐NBPF is a new kind of nonmetal semiconductor with large‐range light absorption characteristics, therefore, application in photodynamic therapy is promising. BP can serve as a photosensitizer to produce^1^O_2_ under 660 nm laser irradiation.^[^ ^65^ ^]^ BP generates singlet oxygen at a quantum yield higher than that of most photosensitizers. In addition, 2D LDHs are also widely used in PDT because of their adjustable size and composition, good stability, and biocompatibility. A recent study discovered that Mg‐Mn‐Al LDH, **Figure** **15**A(a)) with MoS_2_ doping and loaded Ce6(LMM@BSA/Ce6) could efficiently catalyze the decomposing of H_2_O_2_ in the tumor to generate^1^O_2_. The structure of 1,3‐diphenylisobenzofuran alters quickly upon ROS, which was used to indicate the production of^1^O_2_. The absorption peaks of 1,3‐diphenylisobenzofuran at 410 nm with different cumulative times were weakened with 660 nm irradiated, indicating the continuous production of^1^O_2_.^[^ ^297^ ^]^ In addition, researchers used a variety of aromatic phosphorescent molecules in 2D LDHs to prepare supramolecular photosensitizers for achieving long‐lived triplet excitons (Figure 15B(a)).^[^ ^298^ ^]^ The self‐assembled LDHs photosensitizer can be activated at 808 nm irradiation (Figure 15B(b,c)), effectively generating^1^O_2_. In vitro and in vivo experiments have proved that PDT has high efficiency, extremely low toxicity, and good biocompatibility.

2D MOFs have been explored for potential applications in PDT because of their large surface area and porous structures. MOF‐based NBPFs could be prepared by a microwave reactor, and changed the thickness of MOF‐based NBPFs by adjusting the concentration of acid (Figure 15C(a)).^[^ ^112^ ^]^ With the thickness decrease, the surface area and light‐absorption capacity of the 2D MOFs increase because of their large surface area and tunable structures. MOF‐based NBPFs were prepared using a microwave oven and controlled their thickness by adjusting the concentration of acetic acid (Figure 15C(a)).^[^ ^112^ ^]^ With the thickness decreases, the surface area and light‐harvesting capacity of the 2D MOFs enlarge prominently. 2D MOFs have large surface areas and tunable structures,^[^ ^299^ ^]^ which are beneficial to PDT therapy, and thus have broad application prospects in PDT therapy (Figure 15C(b)). The results show that thinner 2D MOFs are more favorable for the generation of^1^O_2_ due to higher quantum yield and higher charge transfer efficiency.

COF‐based NBPFs are porous organic materials and have huge application prospects in the loading of photosensitizers. In 2019, a 2D COF‐based photosensitizer named COF‐909 was prepared by L‐3N inactive molecular, which made its photoexcitation at 630 nm and reactive oxygen species generation.^[^ ^184^ ^]^ COF‐909 showed high phototoxicity in vitro for CT‐26 colon cancer cells leading to 80% of cell death (Figure 15D). However, COFs have a high porosity, which makes for the diffusion of oxygen and the release of ROS in human cells. Furthermore, its excellent photostability and biocompatibility also promoted PDT applications.

Besides, 2D‐NBPFs containing oxidized metals (iron, copper, manganese, platinum, etc.) or disulfide bonds have many advantages in constructing such functional platforms. Especially, 2D‐NBPFs can significantly consume GSH and increase ROS levels in tumor cells, which is beneficial to tumor treatment.

### 4.5 Sonodynamic Therapy

Sonodynamic therapy (SDT), is an emerging and noninvasive therapeutic method that generates ROS. It takes advantage of exogenous ultrasound (US) to trigger sonosensitizers, which have the characteristics of strong focusing and little damage to normal tissue. BP‐based 2D‐NBPFs are widely used in photodynamic and photothermal therapies, but the sono‐sensitizing effects of BP‐based 2D‐NBPFs are still in their infancy. A recent study has shown that BP‐based 2D‐NBPFs as the piezoelectric material can be polarized under the US, and then react with surrounding O_2_ and water molecules to produce ROS. In addition, metal particles can be anchored on its surface to make the surface plasmas resonance and capture excited electrons, thus inhibiting the rapid recombination of electron–hole pairs from enhancing SDT.^[^ ^300^ ^]^

**Figure 16:**
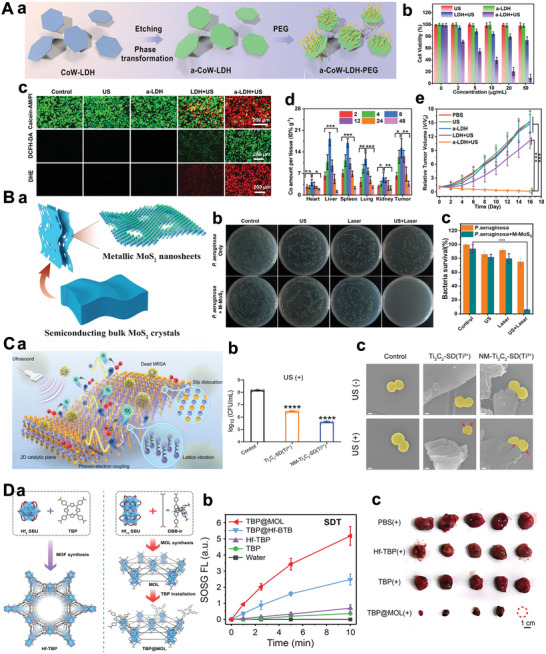
A(a)) Schematic image of a‐CoW‐LDH‐PEG. (b) Cell viability under different conditions. (c) Calcein‐AM/PI, DCFH‐DA, and DHE staining images of cells under different conditions. (d) Biodistribution image of the a‐CoW‐LDH‐PEG in mice (e) Tumor growth curves of 4T1 tumor‐bearing mice. Reproduced with permission.^[^ ^104^ ^]^ Copyright 2023, Wiley‐VCH. B(a)) Illustration image of exfoliation of bulk MoS_2_. (b) Photographs of bacterial colonies formed by *P*. *aureus* (c)  Statistical chart of *P*. *aureus* after treatment with different conditions. Reproduced with permission.^[^ ^301^ ^]^ Copyright 2022, Springer Nature. C(a)) Schematic image of mechanism and antibacterial performance of Ti_3_C_2_. (b) Methicillin‐resistant staphylococcus aureus‐killing abilities of different samples after treatment with the US. (c) SEM images of Methicillin‐resistant staphylococcus aureus on different samples without or with US. Reproduced with permission.^[^ ^302^ ^]^ Copyright 2022, Wiley‐VCH. D(a)) Schematic image of Hf‐TBP and TBP@MOL. (b) ^1^O_2_ generation of various samples upon US. (c) Image of excised tumors of mice after different conditions. Reproduced with permission.^[^ ^113^ ^]^ Copyright 2023, Wiley‐VCH.

Previously, LDH‐based 2D‐NBPFs is rarely used in the field of sonodynamic therapy. However, there has been a report on CoW‐LDH, which can function as high‐efficiency inorganic sonosensitizers for sonodynamic cancer therapy in recent years.^[^ ^104^ ^]^ As shown in **Figure** **16**A(a), the a‐CoW‐LDH nanosheets were prepared via CoW‐LDH under US irradiation. Its SDT performance is ≈5 times better than LDHs, which is attributed to the crystalline‐to‐amorphous phase transformation‐induced bandgap narrowing, electronic structure changing, and defect generation. These are beneficial to isolating of electron–hole pairs and preventing their recombination, thus producing high ROS generation efficiency (Figure 16A(b–e)).

The crystal phase of TMDCs‐based 2D‐NBPFs, which is determined by their atomic arrangements and/or coordination modes, recently, has been demonstrated to have exist an obvious effect on the physicochemical properties. It has been reported that 1T/1T´‐phase could endow 2D MoS_2_ (M‐MoS_2_) with much the defective (Figure 16B(a), thus enhancing the SDT effect compared to the semiconducting 2H‐phase MoS_2_ nanosheets (S‐MoS_2_). In Figure 16B(b,c), M‐MoS_2_ shows an excellent bactericidal effect.^[^ ^301^ ^]^

Ti_3_C_2_, as an MXene‐based 2D‐NBPFs, was usually used for PDT therapy. However, it is rarely used as an SDT treatment. Due to the original Ti_3_C_2_ has no crystal defects and exhibits ultrasonic silence. Therefore, by creating a defect structures on Ti_3_C_2_, the sound‐sensitive performance can be improved. For instance, Yang's group has proved that the defects of specific planar slip dislocations with abundant Ti^3+^ species (Ti_3_C_2_ [Ti_3_C_2_‐SD(Ti^3+^)]), can strongly produce ^1^O_2_ upon the US due to the strong phonon–electron coupling effect. The 2D planar defects have achieved the acceleration of electron transfer and the decrease of the energy barrier to O_2_ surface reaction, thus resulting in massive ^1^O_2_ generation via the reduction reaction (Figure 16C(a–c) ).^[^ ^302^ ^]^

In addition, MOF or COF can also be used as a universal platform to load acoustic sensitizers. As shown in Figure 16D(a), 5,10,15,20‐tetra(p‐benzoate)porphyrin (TBP) sono‐sensitizer was anchored on Hf‐oxo secondary building units (SBUs) of metal‐organic layers (MOLs). In mouse models of cancer, TBP@MOL shows higher SDT efficacy than Hf‐TBP and TBP (Figure 16D(B,C)).

### 4.6 Antipathogen Nanoagents

**Figure 17:**
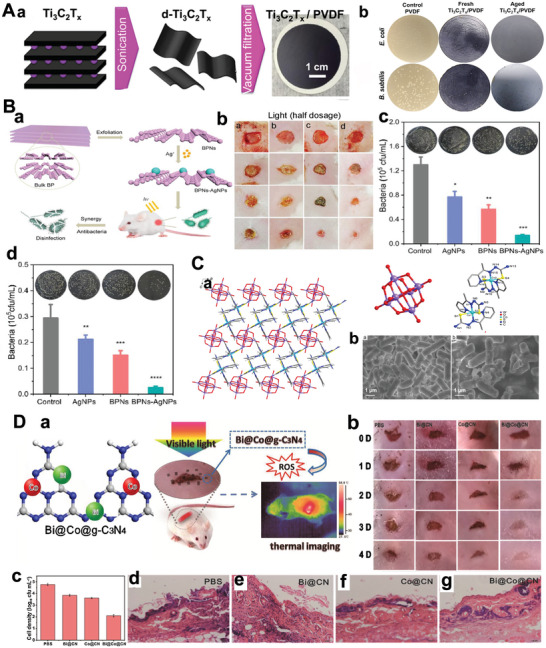
A(a)) Schematic of Ti_3_C_2_T*_x_* membrane fabrication. (b) Images of *E. coli* and *B. subtilis* growth on unmodified polyvinylidene fluoride (PVDF, control), and fresh and aged Ti_3_C_2_T*_x_* MXene coated polyvinylidene fluoride membranes incubated. Reproduced with permission.^[^ ^303^ ^]^ Copyright 2017, Springer Nature. B(a)) The schematic preparation of BPNs–AgNPs based disinfection. (b) TEM characterization of the BPNs–AgNPs. (c) The photographs of *E. coli*‐infected wounds with light irradiation in different treatment groups. (d) The quantified live *E. coli* from the infected wound tissues with (left) and without (right) light. Reproduced with permission.^[^ ^7^ ^]^ Copyright 2020, Wiley‐VCH. C(a)) 2D crystal structure image of polyoxometalates compound. (b) Morphological changes of *E. coli*. Reproduced with permission.^[^ ^309^ ^]^ Copyright 2021, Springer Nature. D(a)) 2D crystal structure of Bi@Co@g‐C_3_N_4_ and schematic diagram of the wound on the mice during the therapeutic process. (b) Using PBS, Bi@CN, Co@CN, and Bi@Co@CN treat mice. (c‐g) Colony growth of wound tissue on agar plates. Reproduced with permission.^[^ ^308^ ^]^ Copyright 2019, Elsevier.

Antimicrobial materials have attracted more and more attention from researchers at home and abroad due to the growing demand for effective antimicrobial strategies. Searching for a new type of antimicrobial NBPFs is very important to take the place of antibiotics due to their usage leading to irreversible drug resistance. A Ti_3_C_2_T*_x_*‐modified membrane was fabricated against *Escherichia coli* (*E. coli*) and  *Bacillus subtilis* (*B. subtilis*),^[^ ^303^ ^]^ which exhibit a superior antibacterial rate (**Figure** **17**A). Therefore, MXene‐based NBPFs would become promising antibacterial materials with internal characteristics, including large specific surface area, light‐induced ROS generation, and excellent photothermal conversion efficiency. In short, 2D MXenes facilitate contact with bacteria and penetrate cell membranes.

In addition, BP also is the most ideal 2D‐NBPF with compelling physiochemical features. It is usually used for eliminating bacteria as a nanofungicide. However, the research on BP‐based antibacterial properties is still in its initial phase.^[^ ^7^ ^]^ Doping silver nanoparticles in BP nanoparticles can enhance their antibacterial ability (Figure 17B(a)). The density functional theory (DFT) calculation demonstrated that BPNs‐AgNPs provide the most suitable active site for facilitating the adsorption and activation of O_2_, leading to the increased production of ROS, which is because of improved electron‐hole separation and recombination of BPNs after AgNP is doped. In addition to enhancing the light‐induced production of ROS, the AgNPs contemporarily contribute to a significantly enhanced affinity toward bacteria. Antibacterial BPNs‐AgNPs contributed to significant wound healing and antibacterial ability (Figure 17B(b, c)). The careful design of this novel 2D hybrid nanomaterial provides a new way to further study the application of BP‐based NBPFs in antibacterial fields.

2D Polyoxometalates, with a variety of structures, high negative charge, and special physical chemistry properties, perform fine biological activities.^[^ ^304^, ^305^ ^]^ Recently, a new 2D polyoxometalates [Co(L)_2_]_2_[W_6_O_19_]_(1)_ was fabricated (Figure 17C(a)),^[^ ^306^ ^]^ which is a new organic–inorganic hybrid compound with high cell penetration ability and chemical stability (Figure 17C(b–d)), as well as high bacteriostatic ability. The experiment has demonstrated that the minimum inhibitory concentrations of *E. coli* and *Staphylococcus aureus* (*S. aureus*) were 0.24 and 0.06 µg mL^−1^, respectively. Hence, the 2D polyoxometalates‐based NBPFs have potential application prospects in biomedicine and offer a possible approach of action for the antibacterial property in the future.

g‐C_3_N_4_‐based NBPFs are a safe and effective antibacterial material with little toxicity and side effects because it generates fewer disinfection by‐products.^[^ ^307^ ^]^ Recently, studies have found that multifunctional nitrogen‐rich carbon‐coated bismuth/cobalt nanoparticles (Bi@Co@CN) with foam‐like structures synthesized by a simple two‐step approach, which severely damage the bacterial cell membrane by verification of experiment.^[^ ^308^ ^]^ Via evaluating the activity of Bi@Co@CN in vivo, the PDT therapy effects and the bactericidal effect of different materials on wound healing in an animal model were explored (Figure 17D(a–g)). It showed that the material is very effective at healing wounds accompanied by bacteria without causing detectable damage to major organs. Therefore, the g‐C_3_N_4_‐based composite material has great potential as a safe multimode photodynamic inactivation and wound healing treatment system. However, due to the inherent disadvantages of g‐C_3_N_4_‐based NBPFs, such as small specific surface area, limited visible light absorption, poor charge transfer properties, and high exciton recombination, its photocatalytic efficiency is reduced. By doping foreign elements in the C_3_N_4_ matrix, it is expected to reduce the bandgap and expand the pathway of visible light absorption, increasing its antibacterial activity.

### 4.7 Tissue Regenerative Scaffolds and Platforms

In the past few years, 2D‐NBPF‐derived materials have risen as emerging stars on the path of researchers searching for ideal materials for regenerative medicine. First, they showed superior mechanical and electrical properties matching the native tissues, which is essential for cell recruiting and proliferation and subsequent tissue regeneration. Second, for stem cell engineering, the planar aromatic structure imparts outstanding performance to immobilize biological molecules. Additionally, the bioactivity of 2D‐NBPFs to cause and guide stem cell differentiation effectively is beneficial to tissue rebuilding.^[^ ^310^ ^]^ Therefore, it is not surprising that 2D‐NBPFs have captured intense interest in regenerative medicine. For example, 2D‐NBPFs have shown superior activities to other materials for neuron regeneration due to their good conductivity.

**Figure 18:**
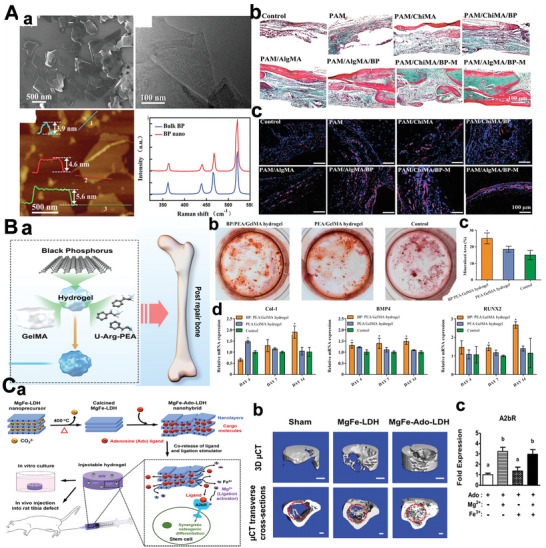
A(a)) The characterization of BP nanosheets, including SEM, TEM, AFM, and Raman spectra. (b) Tissue histology analyses with older trichrome staining. (c) The vascularization of the implanted bone construct was demonstrated via von Willebrand factor immunostaining. Reproduced with permission.^[^ ^311^ ^]^ Copyright 2019, Wiley‐VCH. B(a)) Schematic image of hydrogel@BP for enhancing bone regeneration. (b) Alizarin Red S staining images of hDPSCs. (c)  Data analysis of the mineralized area of a culture dish. (d) Osteogenic gene expression in hDPSCs. Reproduced with permission.^[^ ^9^ ^]^ Copyright 2019, American Chemical Society. C(a)) Preparation scheme of MgFe‐LDH. (b) With the injection of “MgFe‐LDH” or “MgFe‐Ado‐LDH,” 3D microcomputed tomography (µCT) images of bone defects. (c) Quantitative gene expressions of hMSCs for the osteogenic marker (OCN) and A2BR (A2B receptor). Reproduced with permission.^[^ ^105^ ^]^ Copyright 2021, Wiley‐VCH.

BP is often used in PDT and PTT therapy due to its good photoelectric properties. However, it is almost rarely used for post‐trauma tissue regeneration as an electroactive in vivo. Qian et al. synthesized a BP nanoscaffold of axially assembled layer by layer and researched that a 2D BP‐based nanoscaffold could induce angiogenesis and nerve regeneration. These studies provide new insights into the ability of 2D BP‐fabricated 3D scaffolds for neural engineering to provide neural tissue regeneration.^[^ ^312^ ^]^ In order to further improve the biocompatibility of the material novel 2D‐NBPFs consisting of the double network (DN)/nanoengineered (NE) hydrogels and BP nanosheets were synthesized (**Figure** **18**A(a)).^[^ ^93^ ^]^ As shown in Figure 18A(b), there are a larger number of dense and layered connective bones was generated in mineralized NE gels (PAM/ChiMA/BP‐M, PAM/AlgMA/BP‐M) than that of control and PAM gels groups. Moreover, the formed blood vessel was characterized via von Willebrand factor immunostaining in Figure 18A(c), which revealed lots of blood vessel formation within mineralized NE hydrogels, while the control and PAM groups had limited vascularization.^[^ ^311^ ^]^ The results of these experiments confirmed that the BP nanosheet‐wrapped DN hydrogels are biocompatible and can accelerate bone regeneration in vivo. In addition, the researchers have developed a BP nanosheet‐based hydrogel platform for steady and continuous providing phosphorus (Figure 18B(a)). The obtained BPNs are beneficial for improving the mechanical properties of hydrogels and accelerating the mineralization of bone. The principle is that BPNS‐containing hydrogels promote the osteogenic differentiation of human dental pulp stem cells (hDPSCs) by affecting the bone morphogenic protein‐related transcription factor 2 pathway. The results in bone defects indicated that BPNs promote bone regeneration. The results of osteal defective repair indicate that the continuous supply of calcium and phosphorus‐free strategy and this BPNs‐containing hydrogel platform is expected to be effective in bone regeneration.

LDH nanoparticles are one of the 2D‐NBPFs and are often used for tissue and cell regeneration.^[^ ^47^ ^]^

There are assembled from positively charged host laminates and interlayer anions through noncovalent interactions.^[^ ^313^ ^]^ A novel LDH‐based nanocomposite material (MgFe‐Ado‐LDH)^[^ ^105^ ^]^ was prepared (Figure 18C(a)), which activated adenosine A2bR through the dual synergism of adenosine and Mg^2+^/Fe^2+^, thus promoting stem cell Osteogenic differentiation. Furthermore, µCT analyses indicated that the injection of the MgFe‐Ado‐LDH nanocomposite material promoted the healing of rat tibial bone defects via the activation of A2bR (Figure 18C(b,c)).

### 4.8 Cellular and Tissue Imaging

**Figure 19:**
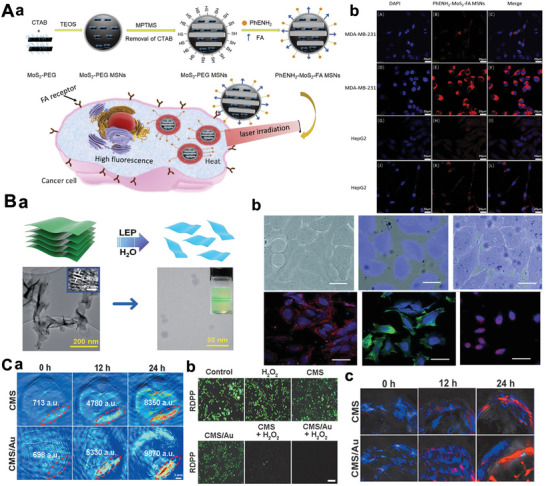
A(a)) Schematic image of the preparation of PhENH_2_‐MoS_2_‐FA MSNs. (b) Confocal laser scanning microscope photos of MDA‐MB‐231 cells and HepG2 cells after cultivation with PhENH_2_‐MoS_2_‐FA MSNs. Reproduced with permission.^[^ ^318^ ^]^ Copyright 2019, Elsevier. B(a)) TEM image of bulk and nanosheets of TTA–DFP. (b) Confocal photos of HeLa cells incubated with exfoliated 2D TTA–DFP. Reproduced with permission.^[^ ^319^ ^]^ Copyright 2018, Royal Society of Chemistry. C(a)) Photoacoustic (PA) images of tumor sites. (b) Fluorescence images of tumors O_2_ releasing detected with RDPP and mean fluorescence intensity. (c) PA images of oxyhemoglobin and hemoglobin in the tumors. Reproduced with permission.^[^ ^320^ ^]^ Copyright 2020, Wiley‐VCH.

Over the past few decades, the advance in nanotechnology has promoted the development of bioimaging and cancer diagnosis. 2D‐NBPFs own unique optical properties due to their tunable bandgap, high quantum yields, broad absorption spectra, and resistance to photobleaching, and they provide an excellent platform for diagnostic imaging. One of the most studied methods is fluorescent imaging, which employs a fluorescent agent to mark the targets. An ideal fluorescent agent requires high quantum yields, good photostability, easy modification with targeting groups, and low toxicity. Conventional tag materials containing heavy metal atoms, like CdS, are excluded due to their high cytotoxicity. 2D‐NBPFs, especially those that show low toxicity, showed potential utilization for fluorescent imaging, and *g*‐C_3_N_4_, BP, TMDCs, and MXenes‐based fluorescent imaging have been developed.^[^ ^126^, ^314^, ^315^, ^316^, ^317^ ^]^ Another advantage of 2D materials‐based imaging is the fluorescence tunability over nanosheet lateral size, thickness, and surface modification. They are now gradually taking the place of organic dyes for bioimaging purposes. Wang et al. prepared silica nanoparticles (MSNs) loading MoS_2_ nanosheets, which were chemically modified by aggregation‐induced emission fluorogenic PhENH_2_ (**Figure** **19A(a)**). As shown in Figure 19A(b), PhENH_2_‐MoS_2_ performs stable fluorescence emission.^[^ ^318^ ^]^ However, fluorescence is rather limited for in vivo diagnosis because the excitation light is not in the range of tissue penetrative NIR window, and clear images cannot be taken because of the tissue noises. Recently, a triazine‐based COF was synthesized by the microwave‐assisted method, which obtained strong photoluminescence performance with a blueshift upon exfoliation in water (Figure 19B(a)). The result has proved that these triazine‐based acid‐stable luminescent triazine‐based COF (TTA–DFP covalent organic nanosheets) enter HeLa cells via clathrin‐intervened endocytosis, selectively accumulating in the nucleus to stain it (Figure 19B(b)).^[^ ^319^ ^]^ Consequently, 2D COF‐based NBPFs could be a prospective biosensor in bio‐imaging applications.

In addition, 2D nanosheets could make loading metal as high attenuation coefficient materials for multimodal imaging and combination therapy. For in vivo imaging, photoacoustic tomography has attracted much recent attention due to its specific features of spatial resolution and high imaging depth, which require special contrast agents to achieve desired imaging performance. The photoacoustic tomography effect is based on generating acoustic waves by a photoabsorbing, preferably NIR‐absorbing material. When endogenous molecules fail to provide enough signals for high‐resolution photoacoustic tomography imaging, contrast agents should be administered to achieve desired imaging performance. X‐ray computed tomography (CT) imaging is a well‐established clinical imaging tool that is noninvasive and offers 3D visualization with high resolution. For CT imaging, a contrast agent to enhance incident X‐rays is required for high‐resolution imaging, where 2D nanosheets with strong X‐ray attenuation can serve well. The presence of heavy elements like Mo, W, Bi, and Ti render 2D nanosheets more powerful contrast agents than graphene‐based nanomaterials. Recently, photoacoustic tomography/CT dual imaging has been achieved by the 2D nanosheets, like WS_2_, MoS_2_, TiS_2_, and Bi_2_Se_3_, whereas the 2D nanosheets are used as a contrast agent for both PAT and CT.^[^ ^195^, ^225^, ^321^ ^]^ WS_2_ nanosheets based mesoporous polydopamine nanosponges (MPDA NSs) materials (MPDA‐WS_2_@MnO_2_) exhibited radio sensitization enhanced behavior.^[^ ^109^ ^]^ The mesoporous structure of mesoporous polydopamine nanosponges (MPDA NSs) provided a reservoir for the self‐assembly of WS_2_ QDs to form MPDA‐WS_2_ NPs, in which WS_2_ QDs support the radiation sensitization effect. The MPDA‐WS_2_@MnO_2_ have been appraised as contrast agents for CT that have the potential for real‐time guidance and monitoring during cancer therapy.

Magnetic resonance imaging (MRI) has a long history of being used clinically, which utilizes a magnetic material as a contrast agent for biomedical imaging. Superparamagnetic iron oxide nanosheets have attracted extensive attention, which can induce localized inhomogeneity of magnetic field and induce a decrease in regional signal intensity due to the shortening of *T*_2_ relaxation time. 2D iron oxide nanoparticles have been successfully used for bioimaging applications. Loading iron oxide on a nanosheet platform can significantly prevent the aggregation of iron oxide nanoparticles in vivo, thereby prolonging circulation time in blood. By constructing Cu_2_MoS_4_/Au heterostructures as MRI imaging agents, which showed excellent computed tomography imaging performance because of its outstanding X‐ray attenuation property of Au element (Figure 19C(a–c)).^[^ ^320^ ^]^ In summary, 2D materials have been shown to be effective diagnostic reagents in nanomedicine. By incorporating functional elements into the lattice and finely controlling the chemical composition, 2D‐NBPFs can support multimodal imaging capabilities and strong particle stability in the organs of living organisms. The synergy between functional elements in 2D‐NBPFs lattices and intercalated or surface‐conjugated molecules can provide enhanced imaging performance.

### 4.9 Biosensing Nanodevices

Biosensing is a significant diagnostic technique spanning various fields, including medical, environmental, drug, and food fields. However, a biosensor with high sensitivity, selectivity, and efficiency is still under pursuit. As emerging 2D‐NBPFs with particular optical, electronic, and magnetic properties, TMDs have been applicated in electrochemical biosensors, impedance biosensors, fluorescence biosensors, as well as biomolecular labels.^[^ ^322^ ^]^ Normally, sensing is achieved through the interaction of the target with the TMDs, which will induce changes in properties, including electrochemical impedance, fluorescence, etc.

**Figure 20:**
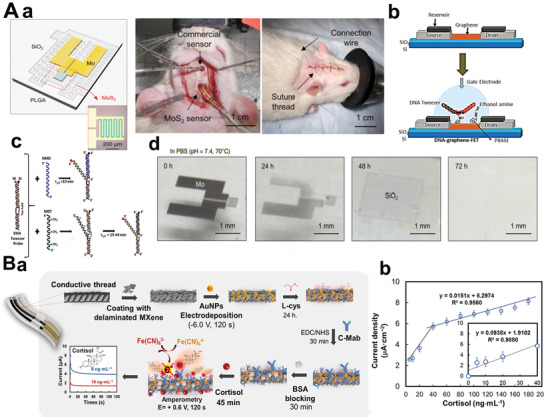
A(a)) Schematic image of MoS_2_‐based transistors biosensor and the images of a MoS_2_ sensor implanted in a rat together with a commercial one. (b,c) Schematic illustration of the DNA tweezer probe conjugation to the graphene field‐effect transistor and strand displacement DNA. (d) Sensing of the MoS_2_‐based FET sensor. Reproduced with permission.^[^ ^327^ ^]^ Copyright 2018, Springer Nature. B(a)) Electrochemical immunosensor for cortisol detection. (b) Calibration plot of the cortisol sensor. Reproduced with permission.^[^ ^328^ ^]^ Copyright 2022, Elsevier.

Compared to graphene‐based sensing platforms, the emerging 2D MoS_2_‐based NBPFs have tunable bandgaps between the semiconductor and insulator states, preventing current leakage and allowing high sensitivity.^[^ ^323^ ^]^ Consequently, in the past few decades, 2D MoS_2_ nanosheets have been developed to detect molecules like K^+^, glutathione, DNA, and many other biological reactions.^[^ ^323^, ^324^, ^325^, ^326^ ^]^ For example, CVD‐grown a thin layer bioabsorbable MoS_2_‐based sensor was synthesized via CVD methods as shown in **Figure** **20**A(a), which could monitor pivotal parameters about the recovery of traumatic brain injury, including intracranial pressure, temperature, motion and strain (Figure 20A(b)). Experiments in vivo mice models demonstrated the superior performance of these sensors with measured intracranial parameters (Figure 20A(c)). This technology plays a vital role in diagnostic/therapeutic functions, pointing out the direction for integrating more 2D‐NBPFs into the sensing platform and clinical applications of biomedical implants.^[^ ^46^, ^327^ ^]^

2D BP‐based NBPFs was also researched as a biosensing platform for monitoring carcinoembryonic antigen labeled in the breast and colon cancer samples. The Au‐modified phosphorene showed excellent catalytic activity for the reduction of 4‐nitrophenol; the signal turns from yellow to white and catalyzes the conversion of 4‐nitrophenol, which can be used for monitoring carcinoembryonic antigen. However, 2D BP is unstable under normal environmental conditions, leading to the limitations of its application. Compared to 2D BP‐based sensing platforms, 2D MXene‐based biosensors are a more stable detection agent for detecting biomolecules because of their outstanding physical and electrical characteristics.

MXene‐based NBPFs can be used as an electrochemical immunosensor to identify effective biomarkers of adrenal disease and detect sweat cortisol (Figure 20B). The principle is that MXene‐based NBPFs increase the specific surface area of the conductive thread electrode, which improves the anticortisol immobilization ability, and makes the sensitivity of the sensor lower. Under the first‐rank conditions, the immunosensor has high sensitivity with a linear range of 5–180 ng mL^−1^ and a detection limit of 0.54 ng mL^−1^. Moreover, this immunosensor offers high reproducibility and long‐term storage stability (≥6 weeks).

In summary, 2D‐NBPFs offer several advanced features, such as monolayer or stacked‐layered structures affording extremely large surface areas, organized and customizable morphologies, design flexibility, and predominant electrical conductivity. These physicochemical properties of 2D‐NBPFs make them a suitable candidate material for biosensing, especially the rapidly developing flexible and wearable devices.

## 5 Future Perspectives and Conclusions

In this review, we comprehensively introduced a multidisciplinary overview of the design principles, synthetic methods, and structure‐characteristic correlation study of recent developments in creating advanced NBPFs via emerging 2D materials. Uniquely, centering around a recent overview of comprehensive strategies for designing 2D‐NBPFs, we perform a cross‐comparison of their strengths and weaknesses. Moreover, we emphatically pay attention to engineering and analyzing the molecularly restructured microenvironments and biofunctionalities of 2D‐NBPFs with well‐defined chemical structures or configurations. Next, this review is summarized in two sections: future outlook and general conclusion.

### 5.1 Future Perspectives

**Scheme 5:**
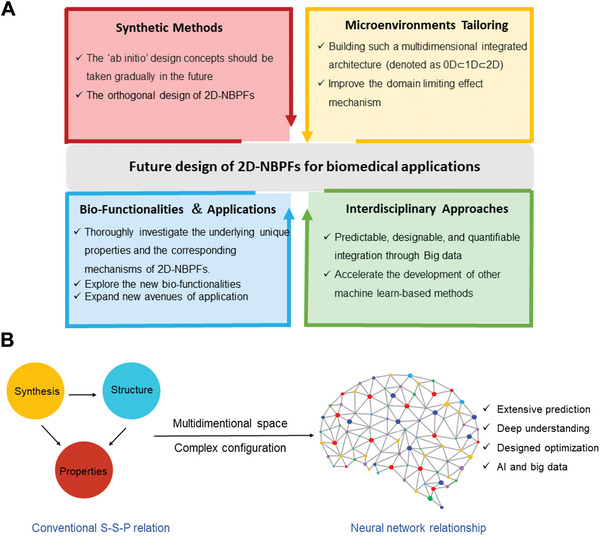
Proposed approaches for developing advanced 2D‐NBPFs in the future.

Clinical applications of 2D materials still face challenges that require innovative designs based on 2D‐NBPFs biological systems. Therefore, some suggestions on future directions, which involve synthetic methods, microenvironments tailoring, biofunctionalities, and interdisciplinary approaches, are given below for the development of 2D‐NBPFs in future advanced therapeutic/diagnostic applications (**Scheme** **5**A).

1) For the synthetic methods of 2D‐NBPFs, most of the synthetic systems on building 2D‐NBPFs are constituted by trial‐and‐error methods. It is proposed that taking the “ab initio” concept will offer abundant new programmed synthetic methods to engineer the chemical structures and coordination environments of 2D‐NBPFs with unique molecularly restructured microenvironments and bio‐functionalities. For instance, the orthogonal design of 2D‐NBPFs could offer great promise for achieving an ideal diagnosis and treatment platform or exhibit advantages in the aspects of biological response and therapeutic applications where integration of various 2D‐NBPFs is essential.
2) For the microenvironments tailoring of 2D‐NBPFs, in addition to the design and analysis of interface microenvironments and biological functions of 2D‐NBPFs, the establishment of spatially constrained environments will also change the optical, electrical, magnetic, and biocatalysis properties of 2D‐NBPFs. For example, molecular orbital energy could be increased by building multidimensional integrated architectures with the advantages of 0D, 1D, and 2D subunits. The method changes the activity of the confined molecule, leading to decreased molecular adsorbent force and enhanced surface reactions, which may be beneficial to the occurrence of enzyme‐catalyzed reactions and improve the biosensing function. However, the current theoretical model of the confinement effect is not perfect, and more accurate theoretical models need to be constructed to quantitatively guide the design and regulation of the physics and microenvironments of 2D‐NBPFs.
3) For the biofunctionalities regulation of 2D‐NBPFs, further exploration of new 2D materials can also provide new avenues for developing 2D‐NBPFs. In addition, the in‐depth study of the potential properties and basic mechanisms of every type of 2D material is beneficial to predict its potential biological effects and provides a favorable way to build a multidisciplinary biomedical all‐around platform. For instance, besides various cancer diagnoses and treatments, 2D materials should be applied to other biomedical fields to treat difficult diseases, such as bone tissue engineering, cardiovascular disease detection, Alzheimer's disease, artificial organ regeneration, etc.
4) For the interdisciplinary approach of exploring 2D‐NBPFs, since Big data and Adobe Illustrator (AI) could change the way material sciences are studied, with access to huge databases of materials, scientists can simply search for compounds with specific properties or characteristics. The fusion of big data and artificial intelligence has helped scientists reveal the underlying relationship between the formation mechanism, atomic structures, and material properties of compounds. Especially, 2D materials with unique physical and chemical properties are widely studied, and everyone is seeking how to understand functional relationships better. However, at present, the exploration of 2D‐NBPFs is limited by the need for numerous complex experiments; therefore, researchers should consider machine learning with Big data and AI as new strategies to accelerate their future development.

In summary, a deep understanding of synthesis–structure–property relationships (S–S–P) will be conducive to creating 2D‐NBPFs for diverse biomedical applications. However, conventional S–S–P relations are excessively complex, which leads to challenges in the design of 2D‐NBPFs. Emerging data‐trained mathematical models may learn and facilitate extensive property prediction, guided optimization, and fundamental understanding of using 2D‐NBPFs for biomedical applications. Such a neural network of multidimensional models may become a new standard for studying complex 2D‐NBPFs (Scheme 5B).

### 5.2 Conclusion

Over the past few years, many works have indicated that presenting the 2D‐NBPFs in the fields of diagnostic and therapeutic applications significantly shows broad prospects.^[^ ^329^ ^]^ Unless offering significant design standards on 2D‐NBPF microenvironments, this review also provides forward‐looking guidance for the future developments of applicable 2D‐NBPFs in clinical fields. Many advantages of diverse 2D‐NBPFs mentioned in this review can be summarized below.

1) For the C_3_N_4_, the introduction of defects and functional groups in C_3_N_4_‐based 2D‐NBPFs will give the material tunable bandgap, conductivity, and catalytic activities^[^ ^330^ ^]^; in some cases, the photoluminescence emission, water solubility, and biocompatibility can also be modified, which are conducive to the wider application of C_3_N_4_ as active nanomaterials for nano‐bio‐interfaces.
2) For the BP, the bandgap of BP‐based 2D‐NBPFs is related to its thickness, which can be adjusted from 0.3 to 2.0 eV by controlling the thickness of BP. The adjustable bandgap indicates the wide absorption of BP in the UV and the whole visible region, which is beneficial for tumor therapy and biosensor applications; this should be further explored to fully disclose the electronic structures of BP via different chemical and physical pathways.
3) For the LDHs, the metal oxide layer in LDHs has a high density of positive charge, and there is electrostatic attraction, hydrogen bonding, and van der Waals forces within and between the anion ion of layers. Therefore, specific drugs can be prepared by anion intercalation or cationic drug loading. Moreover, compared with other 2D nanomaterials, LDHs have been widely researched in the application of biomedicine, especially in the field of drug delivery, due to their low toxicity.^[^ ^146^, ^147^ ^]^
4) For the MXenes, the nano‐bio‐interface properties of MXene‐based 2D‐NBPFs are reliant on the style and make‐up of the M and X sites, as well as the stoichiometry of surface terminations. Additionally, by reducing the *n* value of MXenes, the prominent excitation peaks in their optical spectra shift to higher energies, thus significantly tuning their electronic structures and interface microenvironments. These characteristics indicate that it will be facile to modify the photothermal efficiencies, biocatalytic activities, and biosensing properties.
5) For the TMDCs, the intrinsic properties of TMDCs‐based 2D‐NBPFs can be facilely modulated by their phase structure, defects, heteroatom doping, and heterojunctions. The surface regulation strategies, such as engineering defects or doping heteroatoms, have been reported to be able to enhance the catalytic performance of TMDCs. The heterojunction construction is able to promote electron transfer, thus improving electrical conductivity. Recent studies have shown that TMDCs‐based 2D‐NBPFs can also immobilize many biomolecules per unit area and can be used for efficient biosensor design to detect various analytes.
6) For the MOFs, the MOFs‐based 2D‐NBPFs are endowed with a large specific surface, high porosity, controllable surface functionalization, and excellent biodegradability, which is beneficial to control molecule delivery and release as drug loading substrate. Moreover, these materials also can be developed as biocatalysts to mimic enzymatic activities for biosensing and tumor therapy.
7) For the COFs, the COFs‐based 2D‐NBPFs are made of molecular building blocks linked by reversible chemical bonds, thus making them more susceptible to biodegradation. Furthermore, the designable pore structure and adjustable bandgap make COFs with dominant drug release capability and photothermal or photodynamic properties. Moreover, due to the precise chemical structures of COFs and controllable pore sizes, they can serve as promising candidates for designing biocompatible enzymatic platforms.

Obviously, each 2D‐NBPFs in the field of biomedical therapy filed has its unique advantages. Although 2D‐NBPFs show great potential and have made amazing progress, their further clinical translation still faces a lot of challenges and key issues.^[^ ^8^, ^47^, ^331^, ^332^ ^]^ The long‐term biosafety concern is the major obstacle to the clinical use of 2D‐NBPFs,^[^ ^297^ ^]^ especially those nonbiodegradable ones, which are likely to remain inside the body for a long time. Some recent studies have verified that tuning the chemical components, sizes, and surface structures of 2D‐NBPFs is critical for reducing their retention in normal tissues but enhancing their accumulation in a specific organization (**Table** **4**).

**Table 4:** Biodegradation rate and biodistribution of 2D‐NBPFs

Material	Size & thickness	Modification methods	Main accumulation in organs	Biodegradation‐rate	Refs.
g‐C_3_N_4_	Size: 100 nm Thickness: 1.1 nm	PEG	Liver, lungs, spleen, Heart^[^ ^190^ ^]^	N.A.	[333, 334]
BP	Size: ≈120 nm Thickness: 1–2 nm	PEG, poly(lactic‐*co*‐glycolic acid, polyoxometalates, titanium ligand, exosomes	Liver, lungs, kidneys	Fast	[100, 335, 336, 337, 338]
Mg‐Al‐LDH	Size: ≈50–285 nm Thickness: ≈0.9 nm	Polyglutamic acid, poly(vinyl chloride)	Liver, lungs, kidneys	Fast	[339]
Ti_3_C_2_	Size: ≈150 nm Thickness: ≈0.6 nm	Soybean phospholipid	Liver, lungs, spleen	N.A.	[218]
Ta_4_C_3_	Size: ≈100 nm Thickness: ≈1 nm	Soybean phospholipid	Liver, lungs, spleen, heart	N.A.	[156]
Nb_2_C	Size: ≈150 nm Thickness: 0.3–0.8 nm	Polyvinylpyrrolidone	Liver, lungs, spleen, heart	Fast	[340]
Mo_2_C	Size: ≈143 nm Thickness: 2.2–9.0 nm	Polyvinyl alcohol	Liver, lungs, spleen	Fast	[341]
WS_2_	Size: ≈246 nm Thickness: ≈4 nm	PEG	Liver, lungs, spleen	Slow	[8, 342]
MoS_2_	Size: ≈91 nm	PEG, glucose oxidase	Liver, spleen	Fast	[343, 344]
TaS_2_	Size: ≈143 nm Thickness: 2.2–9.0 nm	PEG	Liver, spleen	N.A.	[345]
TiS_2_	Size: ≈143 nm Thickness: 2.2–9.0 nm	PEG	Liver, spleen	Slow	[346]
FePS_3_	Size: ≈143 nm Thickness: 2.2–9.0 nm	Poly(vinylpyrrolidone)	Spleen, lungs	N.A.	[347]
PPF‐Gd‐MOF	Size: ≈110 nm Thickness: 21.0 ± 9.4 nm	PEG, Polyvinyl Pyrrolidone	Liver, lungs, spleen, Heart	Fast	[348, 349]
COF	Size: ≈90 nm Thickness: ≈1–2 nm	PEG	Liver, spleen	N.A.	[114]

Overall, since we are entering the era of nanomaterials, 2D‐NBPFs can be a versatile platform for a wide range of biomedical usages. However, compared with other nanostructured materials, like carbon nanotubes, metal/metal oxide nanoparticles, polymer micelles, and liposomes, the research of emerging 2D materials at the bio‐nano‐interface is still at an early stage.^[^ ^350^, ^351^, ^352^, ^353^, ^354^, ^355^, ^356^ ^]^ With more and more efforts being devoted to 2D‐NBPFs, it is believed that their potential will be gradually tapped, and inspiring results will be obtained in the near future.

## Conflict of Interest

The authors declare no conflict of interest.
